# Replacement of failed items in a two commodity retrial queueing-inventory system with multi-component demand and vacation interruption

**DOI:** 10.1016/j.heliyon.2024.e24024

**Published:** 2024-01-08

**Authors:** K. Jeganathan, V. Anzen Koffer, K. Lakshmanan, K. Loganathan, Mohamed Abbas, A. Shilpa

**Affiliations:** a Ramanujan Institute for Advanced Study in Mathematics, University of Madras, Chennai, 600005, India; b Department of Mathematics, St. Joseph University, Dimapur, Nagaland, 797115, India; c Department of Mathematics and Statistics, Manipal University Jaipur, Jaipur, 303007, Rajasthan, India; d Electrical Engineering Department, College of Engineering, King Khalid University, Abha 61421, Saudi Arabia; e MLR Institute of Technology, Hyderabad, Telangana, India

**Keywords:** (s,Q) ordering policy, Classical retrial policy, Matrix-geometric solution, Stability condition, Steady state probability vector

## Abstract

This study investigates a crucial aspect of inventory management, which is the process of replacing failed items. In dynamic commercial environments, it is essential to efficiently and strategically replace failed items to maintain operational efficiency and ensure profitability. We consider a two-commodity retrial queueing-inventory system with vacation interruption. Upon purchasing the first commodity, the second commodity is provided as a complimentary item. In contrast, no item is given as a complimentary for the purchase of the second item. Only the first commodity is stored in a dedicated pooled storage for replacement when it fails. The (s,Q) policy governs replenishing the first commodity while the second is replenished through instantaneous ordering. The model considers the multi-component demand rate for customer arrivals. Server vacations are initiated during customer absence in waiting hall or when the first commodity is unavailable. We formulate a level-dependent quasi-birth-and-death process, and its steady-state probability vector is computed using Neuts and Rao's truncation method. The stability condition for the system is derived, and various system performance measures, including expected total cost, number of replaceable items, and customers in the waiting hall and orbit, are established. The comparative analysis between the system with replacement is done with the regular model without replacement, which revealed the efficiency of replacement. The analysis of multi-component demand towards homogeneous arrival highlights the impact of multi-component demand on boosting customer arrival. Also, parametric sensitivity analysis has been conducted numerically over total cost, mean number of failed items for replacement, and mean number of customers in the waiting hall and orbit.

## 1 Introduction

**Figure 1:**
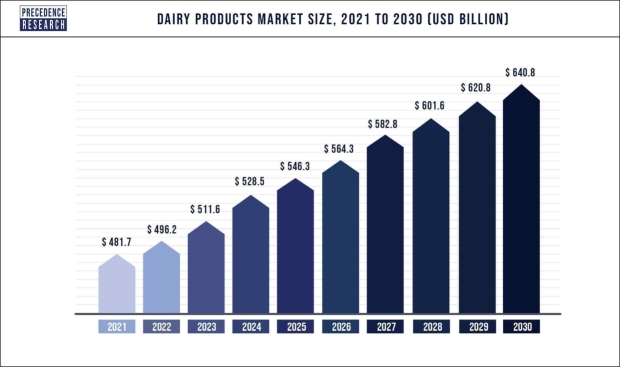
Market size of dairy products, 2021-2023.

The effective management of perishable stocks is paramount in the dynamic environments of the food, chemical, and pharmaceutical industries, serving as a fundamental element of operational success. The fundamental difficulty of these commodities necessitates a certain degree of skill in their preservation and distribution, requiring a deep understanding of the complicated relationship that characterizes inventory dynamics. Therefore, it is not unexpected that academic research has directed its attention towards the intricate realm of mathematical modeling, shedding light on the means to achieve expertise in effectively navigating these pivotal sectors. Dairy products, due to their perishable nature, deteriorate at a rather rapid rate. The surge in demand for these commodities is being shaped by many causes, including expanding the worldwide populace, augmented consumer discretionary income, an amplified focus on wellness, an upswing in milk output in emerging economies, and a rising inclination for protein-laden sustenance. Based on research conducted by Precedence Research [1], it is reported that the global dairy products market had a valuation of $481.7 billion in the year 2021, with a predicted growth to reach $640.8 billion by the year 2030. The projected expansion is expected to be propelled by a significant CAGR of 3.2% throughout the period from 2022 to 2030, which is given in Fig. 1. This dairy product market data is just an example of a perishable item's significance in the market, which shows us the importance of doing mathematical modeling in the perishable inventory system.

### 1.1 Motivation

One of the author's real-life experiences in the Vasanth & Co retail store in Chennai, Tamilnadu, motivated us to apply the replacement idea in our paper. When the author went to inquire about the details of the computer, he observed that during the replenishment of new computers, the repaired monitors were replaced. It is standard practice for the supplier to replace the faulty components at no extra cost. These faulty items are kept separately and are swapped out during the subsequent replenishment cycle. So, we apply the idea of replacing failed items in the stochastic inventory modeling with a service facility.

### 1.2 Literature review

In recent decades, the exploration of perishable inventory systems has captivated the attention of researchers, sparking a surge of interest in unraveling the intricacies of this dynamic field. Numerous notable review articles, such as those by Nahmias [2], Raafat [3], Shah and Shah [4], Goyal and Giri [5], and Karaesmen et al. [6], present comprehensive overviews of various modeling endeavors. For more recent insights, Nahmias [7], Bakker et al. [8], and Janssen et al. [9] offer valuable reviews. These models primarily focus on periodic review systems with fixed lifetimes or continuous review systems with instantaneous reorder supply.

Continuous reviews of perishable inventory models that involve items with random lifetimes have been predominantly studied under the assumption of instantaneous order supply. Notable examples of such studies include the works of Liu and Shi [10], Lian and Liu [11], and Gürler and Özkaya [12]. However, considering positive lead time adds significant complexity to the analyses of these models. The following works deal with the positive lead time.

Sivakumar et al. [13] investigated the joint replenishment policy in a two-commodity inventory system, wherein both commodities are subject to perishability. Manuel et al. [14] examined the (s,S) perishable inventory system with retrial demands, taking into account a random service time that follows an exponential distribution. Additionally, they assumed that the occurrence of arrivals followed a Markovian arrival process. Sivakumar [15] conducted a pioneering investigation into the occurrence of demand from a finite source in a perishable inventory system that incorporated a retrial facility. Notably, the study did not make any assumptions regarding the service facility. In a two-commodity perishable inventory system, Yadavalli et al. [16] considered three different types of customers: Type 1 customers require one unit of the first commodity, type 2 customers require multiple units of the second commodity in bulk, and type 3 customers require one unit of the first commodity and multiple units of the second commodity in bulk.

Koroliuk et al. [17] examined the concept of server vacations within a single server queueing system involving perishable items, in which the server strategically takes a vacation during periods of zero customer presence in the queue. Hanukov et al. [18] proposed incorporating a perishable queueing-inventory model with two independent service stages. Preliminary services are produced in advance, deteriorating over time, and used to reduce future customers' service and wait times. Jeganathan et al. [19] studied comparing a perishable inventory system's two heterogeneous servers with homogeneous servers, leading to the identification of an optimized total cost in the heterogeneous system. In the perishable inventory models, Jeganathan and Reiyas [20] considered two servers exclusively for high and low-priority servers, respectively, with working vacation for the first server and delayed working vacation for the second one. Melikov et al. [21] investigated a perishable queueing-inventory system (QIS) involving fluctuating and fixed order quantities. In contrast to the other models, customers can enter the system even during a stock-out situation.

Barron [22] investigated a perishable inventory system with a demand rate dependent on stock levels and effectively derived the joint probability density function, leading to the attainment of the minimum total cost, by employing the supplementary variable technique. Randhamani et al. [23] studied the optimal ordering policy in a perishable queueing-inventory model, considering scenarios with multiple vacation periods. Through numerical analysis, they found that the varying ordering policy demonstrated greater efficiency when compared to the traditional (s,S) and (s,Q) policies. Jeganathan et al. [24] investigated a QIS that caters to two customer classes, high priority, and low priority, using the discretionary priority discipline. The service process for low-priority customers involves two stages: a preliminary service in stage I and the main service in stage II. On the other hand, high-priority customers only need the main service without any preliminary stage. Anbazhagan et al. [25] obtained a convexity for the total cost rate in a perishable two-commodity system with MAP arrival. The customer can purchase any one of the items or get service only from the system.

Islam et al. [26] presented a model for examining the continuous review inventory policy applied to perishable products with varying demand levels and positive service time in a Jackson queueing network. The system comprises two separate single server nodes, each acting as a queue with its inventory management. Sugapriya et al. [27] compared (s,S) and (s,Q) ordering policies in a perishable QIS with stock-dependent demands. The service rate varies based on the customer level in the queue up to a fixed threshold level and is fixed from the threshold level. The (s,S) attains a minimal total cost than the other one in numerical comparison.

Recently, many models have been studied in the supply chain (SC). Abbasi and Choukolaei [28] provided a comprehensive literature review on the green supply chain design network and analyzed their carbon emission regulation. They showed that carbon cap, cap-and-trade, and carbon tax policies highly influenced the design of SCs. Abbasi and Erdebilli [29] studied the closed-loop supply chain model and assessed the influence of SC actions on optimization, focusing on carbon policies: tax, cap-and-trade, and strict caps. Through statistical testing, they evaluated how these regulations affect total cost and CO_2_ emissions in the SC, and it was shown that the carbon cap policy enforced rigorous limitations on emissions. Emrouznejad et al. [30] presented a review of the literature on Supply Chain Risk (SCR) that investigates the general elements linked to significant themes and patterns in SCR management. It encompasses the process of identifying and evaluating risks, devising methods to mitigate these risks, and analyzing the impact of developing technologies on SCR management. Gonzalez et al. [31] presented a model for managing aggregate production planning in an SC Network that spans multiple products and periods. The different types of health care and sustainable SCs are studied in [32], [33], [34], [35]. For the real-life case study during the COVID-19 pandemic situation on various SCs, one can refer [36], [37], [38], [39].

In all the above papers, authors assume that perished items are promptly discarded. However, only a few papers have examined failed item replacements in QISs. Sivakumar and Anbazhagan [40] investigated a perpetual inventory system handling replaceable items with a base stock policy. Saranya and Lawrence [41] studied the replacement of perished items in a finite stochastic inventory system, allotting a separate storage place. They offered a postponed option for those customers who enter the system while inventory is unavailable. The life of an item and the lead time were considered to follow an exponential distribution. Later, they extended their work in [42], considering the arrival of customers based on MAP and phase type distribution for an item's lead time and lifetime. Malakar and Sen [43] studied the partial replacement of deteriorated items to reduce the total cost. In these works, items that failed while in the storage space are replaced. Saranya et al. [44] considered two types of inventories consisting of new items in one and refurbished items in another with two types of customers. The first type of customers comes to purchase either of these items. However, the second type of customer approach is replacing the failed item with a new one.

### 1.3 Research gap

From Table 1 and the existing literature review, it has been observed that there is an absence of research conducted on replacing failed products in two-commodity QIS with the combination of multi-component demand, retrial facility, and vacation interruption. Therefore, this paper fills that research gap and contribute valuable insights to this field.

**Table 1:** The position of the present research about the previous related works.

References	Two-commodity	Retrial facility	Stock-dependent arrival	Replacement	Vacation
[25]	✓				
[22]			✓		
[26]	✓				
[20]					✓
[19]		✓			✓
[24]		✓			
[17]					✓
[43]				✓	
[14]		✓			
[23]					✓
[41]				✓	
[42]				✓	
[44]				✓	
[15]		✓			
[40]				✓	
[13]	✓				
[27]			✓		
[16]	✓				

This Research	✓	✓	✓	✓	✓

### 1.4 Contributions of the work

• The model considers a two-commodity QIS with the replacement of failed items and retrial facility.
• In this paper, we provide a generalized version of the stock-dependent arrival pattern.
• This paper numerically compares the model's performance with and without the replacement of failed items in the expected total cost.
• The impact of homogeneous arrival rate and multi-component demand is analyzed.
• The sensitivity analysis on the expected number of failed items, customers in the waiting hall, and orbit is also carried out.

### 1.5 Highlights

• Replacement of failed items in the two-commodity QIS has been studied with multi-component demand and retrial facility.
• The joint probability distribution of the system has been computed using Neuts and Rao's truncation method, and the stability condition is derived.
• Comparison on replacement of failed items with the regular model without the replacement shows the efficiency of the replacement facility in the model.
• The multi-component demand rate encourages more customer arrival to the system, and it controls the number of failed items.

The forthcoming sections of this article are structured as follows: Section 2 presents a comprehensive outline of the model's assumptions. Section 3 is dedicated to the analysis and presentation of results. The computation of the steady-state probability vector is addressed in Section 4. Section 5 focuses on deriving various system performance measures. Section 6 involves the numerical analysis of the model. In Section 7, managerial implications and insights are provided. Lastly, the article concludes with the final section summarizing the model's findings, limitations and future works.

## 2 Model explanation

In this segment, we provide fundamental definitions, assumptions of the model, and its respective notations.

### 2.1 Basic definitions

Definition 2.1 **Two component Demand [45]**

The available inventory influences the demand rate until it reaches a threshold $S_{0}$, which remains constant. The demand rate R(i) of the item, at an on-hand inventory level of *i*, is represented in the following format.

$$R(i)=\left\{ \begin{array}{cc} \alpha i^{\beta },\; & \;\text{if}\;i\in \{S_{0},S_{0}+1,\ldots ,S\}\\ D,\; & \;\text{if}\;i\in \{0,1,2,\ldots ,S_{0}\} \end{array}\right.$$

where α,D>0 and 0<β<1 scaling parameter.

Definition 2.2 **Multi-Component Demand Rate for Primary and Retrial Customers**

The multi-component demand rate is the generalized version of two-component demand rate and stock-dependent arrival rate. Let $I_{j}$ be the sets defined by

$$I_{j}=\{1,2,\ldots k_{j}\}\;\;\text{for}\;\;j=1\;I_{j}=\{k_{j-1}+1,k_{j-1}+2,\ldots ,k_{j}\}\;\;\text{for}\;\;j=2,3,\ldots ,c-1\;I_{j}=\{k_{j-1}+1,k_{j-1}+2,\ldots ,S_{1}\}\;\;\text{for}\;\;j=c$$

where $k_{j}$ is a finite positive integer. When the customers enter the waiting hall, the arrival rate of customers is defined as $\lambda_{j}$ for j=1,2,…,c if the present number of primary items (commodity-1) in the system $r_{2}\in I_{j}$ and $\lambda_{0}$ during stock-out period and $\lambda_{0}\leq \lambda_{1}\leq \lambda_{2}\ldots \leq \lambda_{c}$, which is defined as multi-component demand rate. The mean arrival rate $\lambda_{j}$ is defined as

$$\lambda_{j}=\left\{ \begin{array}{cc} \lambda j^{a_{1}},\; & \;\text{for}\;j\in \{1,2,\ldots ,c\}\\ \lambda_{0},\; & \;\text{for}\;j=0 \end{array}\right.$$

where $0\leq a_{1}\leq 1$, λ>0, and $a_{1}$ is scaling factor.

Similarly, the mean retrial rate $\theta_{j}$ is defined as

$$\theta_{j}=\left\{ \begin{array}{cc} \theta j^{b_{1}},\; & \;\text{for}\;j\in \{1,2,\ldots ,c\}\\ \theta_{0},\; & \;\text{for}\;j=0 \end{array}\right.$$

where $0\leq b_{1}\leq 1$, θ>0, $b_{1}$ is scaling factor and $\theta_{0}\leq \theta_{1}\leq \theta_{2}\ldots \leq \theta_{c}$.

Note Suppose that $c=S_{1}$, then the arrival rate becomes $\lambda_{j}$ for all $j=1,\;2,\ldots ,S_{1}$ which is called stock-dependent demand rate.

Definition 2.3 **Replacement Policy for the Perishable Items [42]**

When the primary item's level reaches a predetermined threshold *s*, an order is initiated for a quantity of $Q=(S_{1}-s)-k$ items, where *k* can take on values from 0 to $S_{1}-s$. This order ensures the replenishment of perishable items, which are replaced at no additional cost upon receipt.

### 2.2 Model description

Consider a retrial QIS containing two products, the primary item (commodity-1) and complimentary item (commodity-2), with a finite waiting hall. In this considered system, both commodities are perishable. The maximum capacity commodity-1 is $S_{1}$, and commodity-2 is $S_{2}$. When an item perishes, it is considered to be a failed item. The first commodity is stored in a separate pooled place to replace it when it perishes (failures). However, commodity-2 is not replaced. The perishable rate of commodity-1 and commodity-2 are $\gamma_{1}$ and $\gamma_{2}$, respectively, and the lifetime of each commodity follows an independent exponential distribution. Any arriving customers are offered two kinds of purchasing options. When a customer purchases commodity-1 compulsorily, a second commodity is given as a compliment. However, no complimentary item is given to customers while purchasing the commodity-2.

The primary customers arrive at the waiting hall according to the multi-component demand defined in Definition 2.2 depending upon the first commodity with the rate of $\lambda_{j}$ for j=0,1,2,…,c. The customers cannot be allowed to enter the waiting hall if the waiting hall is full. In this case, they may enter the virtual waiting place called orbit with the probability *r* or leave the system with the probability of 1−r. The arrival of a customer follows non-homogeneous Poisson process with the rate $\lambda_{j}$ for j=0,1,2,…,c and $\lambda_{0}\leq \lambda_{1}\leq \lambda_{2}\ldots \leq \lambda_{c}$. The orbital customers always try to enter the waiting hall to fulfill their demands. The customer in orbit enters the waiting hall whenever there exists at least one space in the waiting hall with the rate of $\theta_{j}$ for j=0,1,2,…,c as defined in the Definition 2.2. The time gap between the arrivals of two successive orbital customers follows an exponential distribution and $\theta_{0}\leq \theta_{1}\leq \theta_{2}\ldots \leq \theta_{c}$. The classical retrial policy is adopted in the retrial process.

In this model, the server operates in two distinct modes: regular and working vacation modes. In the regular mode, the server is always busy. Depending upon the customers' needs, the server provides three different services with rate $\mu_{k}$ for k=1,2,3 with $\mu_{1}<\mu_{2}<\mu_{3}$. Suppose a customer demands the first item At each service completion in the regular mode. In that case, customer departures occur in four different ways:

• If a customer purchases the first commodity with probability $p_{1}$, the departure rate is given by $p_{1}\mu_{1}$.
• If a customer purchases the second commodity with probability $p_{2}$, the departure rate is given by $p_{2}\mu_{2}$.
• If a customer leaves the system after receiving only the service (customers do not purchase anything) with probability $p_{3}$, the departure rate is defined as $p_{3}\mu_{3}$.
• If a customer joins the orbit after receiving only the service (customers do not purchase anything) with probability $p_{4}$, the departure rate is defined as $p_{4}\mu_{3}$.

Where $p_{i}$ represent probabilities, and their sum equals 1.

The server takes a vacation when the primary item is unavailable, or there are no customers in the waiting hall. Suppose a situation exists such that the server becomes busy during the vacation. In that case, the server continues to work at a slower rate $\mu_{k}^{\prime }$ and $\mu_{k}^{\prime }<\mu_{k}$. Similarly, customer departure rates during the server working vacation mode occur in four ways in the same manner in working vacation. While completing the vacation, if there is no customer in the waiting hall or no primary item, the server retakes one more vacation. Upon completion of vacation, if the number of customers in the waiting hall is up to the threshold level *n*, and there is a positive inventory of commodity-1, the server can either switch back to the regular working mode or continue with the vacation with probabilities *q* and 1−q, respectively. The vacation is allowed to the server up to a threshold level *n*. The server cannot continue the vacation when the first commodity is positive and at least one customer is waiting in the hall, with a number exceeding the threshold level *n* by the end of service. In such cases, he is compulsorily interrupted from the vacation to the regular mode. The duration of vacation follows an exponential distribution with the rate *η*.

**Figure 2:**
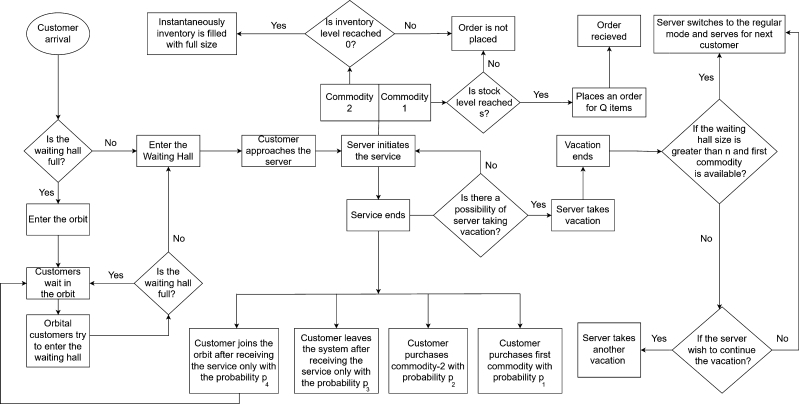
Flow chart of the model.

The depletion of products occurs either through consumption by customers or perishable. Two different reordering policies do the replenishment of each product: the primary item is replenished under the (s,Q) reordering policy with the rate of *β*, which is distributed exponentially, whereas the complimentary item is replenished under the instantaneous reordering policy. When the number of primary items hits *s*, the number of failed items in the pooled storage is replaced according to Definition 2.3. The model is illustrated as a flow chart and is presented in Fig. 2.

Remarks

• Assume that c=1 with $\lambda_{0}=\lambda_{1}$, then the arrival pattern of customers in the model follows the homogeneous arrival pattern.
• Assume that c=1 with $\lambda_{0}\neq \lambda_{1}$, then the arrival rate of customers is turned into the two-component demand rate.
• Suppose that $c=S_{1}$, then the arrival process of the QIS turns into stock-dependent arrival.
• When the probability value r=1, the customer lost is strictly not allowed, so that the primary customers entering into orbit is certain when the waiting hall is fully occupied. Similarly, the new arriving customer will not enter into the orbit when the waiting hall is full, while r=0.

### 2.3 Notations

In this subsection, we provide the notations that has been used in this paper.

$$G_{i}^{j}=\{i,i+1,i+2,\ldots ,j\}H(x)=\left\{ \begin{array}{cc} 1 & x\geq 0\\ 0 & x<0 \end{array}\right.\;\delta_{ij}=\left\{ \begin{array}{cc} 1 & i=j\\ 0 & i\neq j \end{array}\right.\;{\overset{¯}{\delta }}_{ij}=\left\{ \begin{array}{cc} 0 & i=j\\ 1 & i\neq j \end{array}\right.\;\text{0}\;\text{- Zero Matrix of appropriate order.}\;\text{e}\;\text{- Column Matrix of appropriate order with entries 1.}\;N\text{ - Set of all natural numbers.}$$

## 3 Analysis of the model and results

In this section, we examine the construction of the infinitesimal generator matrix to the Markov chain and its transition matrices. Furthermore, we establish the stability condition for the system.

Consider the following random variables at time $R_{1}(t),\;R_{2}(t),\;R_{3}(t),\;R_{4}(t),\;R_{5}(t)$ and $R_{6}(t)$, representing the number of customers in the orbit at time *t*, number of primary items at time *t*, number of failed items in the pooled space at time *t*, number of complimentary items at time *t*, server status at time *t* where

$$R_{5}(t)=\left\{ \begin{array}{cc} 0, & \text{server is on working vacation mode}\\ 1, & \text{server is on regular mode} \end{array}\right.,$$

and number of customers in the waiting hall at time *t* respectively. So the collection $\left\{R(t):t\geq 0\}=\{(R_{1}(t),R_{2}(t),R_{3}(t),R_{4}(t),R_{5}(t),R_{6}(t)):t\geq 0\right\}$ constitutes a six-dimensional level dependent quasi-birth-and-death (QBD) process which is a continuous time Markov process with the state space $E=\overset{5}{\underset{i=1}{⋃}}E_{i}$ where

$$E_{1}=\{(r_{1},r_{2},r_{3},r_{4},r_{5},r_{6})|r_{1}\in N,r_{2}\in G_{0}^{s},r_{3}\in G_{0}^{s-r_{2}},r_{4}\in G_{1}^{S_{2}},r_{5}=0,r_{6}\in G_{0}^{N}\}E_{2}=\{(r_{1},r_{2},r_{3},r_{4},r_{5},r_{6})|r_{1}\in N,r_{2}\in G_{1}^{s},r_{3}\in G_{0}^{s-r_{2}},r_{4}\in G_{1}^{S_{2}},r_{5}=1,r_{6}\in G_{1}^{N}\}E_{3}=\{(r_{1},r_{2},r_{3},r_{4},r_{5},r_{6})|r_{1}\in N,r_{2}\in G_{s+1}^{S_{1}},r_{3}\in G_{0}^{S_{1}-r_{2}},r_{4}\in G_{1}^{S_{2}},r_{5}=0,r_{6}\in G_{0}^{N}\}E_{4}=\{(r_{1},r_{2},r_{3},r_{4},r_{5},r_{6})|r_{1}\in N,r_{2}\in G_{s+1}^{S_{1}},r_{3}\in G_{0}^{S_{1}-r_{2}},r_{4}\in G_{1}^{S_{2}},r_{5}=1,r_{6}\in G_{1}^{N}\}$$

### 3.1 Construction of transition matrices

Due to the arrangement of random variables in the system, {R(t):t≥0} has the infinitesimal generator matrix *P* of the form

(1) P=0123…0(P0,0P0,1)1P1,0P1,1P0,12P2,1P2,2P0,1⋮⋱⋱⋱.

$P_{0,1}$ matrix represents transitions due to customers entering into the orbit.

$$P_{0,1}=\left\{ \begin{array}{cc} V_{r_{2}}, & r_{2}^{\prime }=r_{2},\;r_{2}=0,1,2,\ldots ,S_{1}\\ \text{0}, & \text{otherwise} \end{array}\right.\;V_{r_{2}}=\left\{ \begin{array}{cc} V_{r_{2}}^{\prime }, & r_{3}^{\prime }=r_{3},\;r_{3}=0,1,2,\ldots ,s-r_{2}\;\text{if}\;r_{2}\in G_{0}^{s}\\ & r_{3}^{\prime }=r_{3},\;r_{3}=0,1,2,\ldots ,S_{1}-r_{2}\;\text{if}\;r_{2}\in G_{s+1}^{S_{1}}\\ \text{0}, & \text{otherwise} \end{array}\right.\;V_{r_{2}}^{\prime }=\left\{ \begin{array}{cc} V_{r_{2}}^{\prime \prime }, & r_{4}^{\prime }=r_{4},\;r_{4}=1,2,\ldots ,S_{2}\\ \text{0}, & \text{otherwise} \end{array}\right..$$

For $r_{2}=0$,

$$V_{r_{2}}^{\prime \prime }=\left\{ \begin{array}{cc} r\lambda_{0}, & r_{5}^{\prime }=r_{5},r_{6}^{\prime }=r_{6};r_{5}=0,r_{6}=N\\ p_{4}\mu_{3}^{\prime }, & r_{5}^{\prime }=r_{5},r_{6}^{\prime }=r_{6}-1;r_{5}=0,r_{6}=1,2,\ldots ,N\\ 0, & \text{otherwise} \end{array}\right..$$

For $r_{2}\neq 0$,

$$V_{r_{2}}^{\prime \prime }=\left\{ \begin{array}{cc} r\lambda_{j}, & r_{5}^{\prime }=r_{5},r_{6}^{\prime }=r_{6};r_{5}=0,1,r_{6}=N\\ p_{4}\mu_{3}^{\prime }, & r_{5}^{\prime }=r_{5},r_{6}^{\prime }=r_{6}-1;r_{5}=0,r_{6}=1,2,\ldots ,n+1\\ & r_{5}^{\prime }=r_{5}+1,r_{6}^{\prime }=r_{6}-1;r_{5}=0,r_{6}=n+2,n+3,\ldots ,N\\ p_{4}\mu_{3}, & r_{5}^{\prime }=r_{5}-1,r_{6}^{\prime }=r_{6}-1;r_{5}=1,r_{6}=1\\ & r_{5}^{\prime }=r_{5},r_{6}^{\prime }=r_{6}-1;r_{5}=1,r_{6}=2,3,\ldots ,N\\ 0, & \text{otherwise} \end{array}\right.$$

where j=1,2,…,c and $r_{2}\in I_{j}$.

The matrices $P_{r_{1},r_{1}-1}$ for $r_{1}=1,2,\ldots$ represent the transitions of customers entering into the waiting hall from the orbit. Whenever there exist space fewer than the maximum capacity of the waiting hall, orbital customer enters into the waiting hall.

$$P_{r_{1},r_{1}-1}=\left\{ \begin{array}{cc} W_{r_{1},r_{2}}, & r_{2}^{\prime }=r_{2},\;r_{2}=0,1,\ldots ,S_{1}\\ \text{0}, & \text{otherwise} \end{array}\right.$$

$$W_{r_{1},r_{2}}=\left\{ \begin{array}{cc} W_{r_{1},r_{2}}^{\prime }, & r_{3}^{\prime }=r_{3},\;r_{3}=0,1,2,\ldots ,s-r_{2}\;\;\;\text{if}\;r_{2}\in G_{0}^{s}\\ & r_{3}^{\prime }=r_{3},\;r_{3}=0,1,2,\ldots ,S_{1}-r_{2}\;\text{if}\;r_{2}\in G_{s+1}^{S_{1}}\\ \text{0}, & \text{otherwise} \end{array}\right.$$

$$W_{r_{1},r_{2}}^{\prime }=\left\{ \begin{array}{cc} W_{r_{1},r_{2}}^{\prime \prime }, & r_{4}^{\prime }=r_{4},\;r_{4}=1,2,\ldots ,S_{2}\\ \text{0}, & \text{otherwise} \end{array}\right..$$

For $r_{2}=0$,

$$W_{r_{1},r_{2}}^{\prime \prime }=\left\{ \begin{array}{cc} r_{1}\theta_{0}, & r_{5}^{\prime }=r_{5},r_{6}^{\prime }=r_{6}+1;r_{5}=0,r_{6}=0,1,2,\ldots ,N-1\\ 0, & \text{otherwise} \end{array}\right..$$

For $r_{2}\neq 0$,

$$W_{r_{1},r_{2}}^{\prime \prime }=\left\{ \begin{array}{cc} r_{1}\theta_{j}, & r_{5}^{\prime }=r_{5},r_{6}^{\prime }=r_{6}+1;r_{5}=0,r_{6}=0\\ & r_{5}^{\prime }=r_{5},r_{6}^{\prime }=r_{6}+1;r_{5}=0,1,r_{6}=1,2,\ldots ,N-1\\ 0, & \text{otherwise} \end{array}\right.$$

where j=1,2,…,c and $r_{2}\in I_{j}$.

The matrices $P_{r_{1},r_{1}}$ for $r_{1}=0,1,2,\ldots$ represent the transitions of primary customers entering into the waiting hall, service, vacation completion, replenishment of items and deterioration.

$$P_{r_{1},r_{1}}=\left\{ \begin{array}{cc} X_{r_{1},r_{2}}, & r_{2}^{\prime }=r_{2},r_{2}=0,1,\ldots S_{1}\\ Y_{r_{2}}, & r_{2}^{\prime }=r_{2}-1,r_{2}=1,2,\ldots S_{1}\\ Z_{r_{2}}, & r_{2}^{\prime }=r_{2}+Q,r_{2}=0,1,\ldots s\\ \text{0} & \text{otherwise} \end{array}\right.$$

For $r_{2}=0$,

$$Z_{r_{2}}=\left\{ \begin{array}{cc} Z_{r_{2}}^{\prime } & r_{3}^{\prime }=r_{3},\;r_{3}=0,1,\ldots s\\ \text{0} & \text{otherwise} \end{array}\right.$$

$$Z_{r_{2}}^{\prime }=\left\{ \begin{array}{cc} Z_{r_{2}}^{\prime \prime }, & r_{4}^{\prime }=r_{4},\;r_{4}=1,2,\ldots S_{2}\\ \text{0}, & \text{otherwise} \end{array}\right.$$

$$Z_{r_{2}}^{\prime \prime }=\left\{ \begin{array}{cc} \beta , & r_{5}^{\prime }=r_{5},r_{6}=r_{6}^{\prime };r_{5}=0,r_{6}=0,1,\ldots ,N\\ 0, & \text{otherwise} \end{array}\right..$$

For $r_{2}=1,2,\ldots ,s$,

$$Z_{r_{2}}=\beta I_{\left(s+1-r_{2})S_{2}(2N+1\right)}.$$

For $r_{2}=1$,

$$Y_{r_{2}}=\left\{ \begin{array}{cc} Y_{r_{2}}^{\prime }, & r_{3}^{\prime }=r_{3},r_{3}=0,1,2,\ldots ,s-1\\ Y_{r_{2}}^{\prime \prime }, & r_{3}^{\prime }=r_{3}+1,r_{3}=0,1,2,\ldots ,s-1\\ \text{0}, & \text{otherwise} \end{array}\right.$$

$$Y_{r_{2}}^{\prime }=\left\{ \begin{array}{cc} Y_{r_{3}}^{\prime \prime \prime }, & r_{4}^{\prime }=S_{2},r_{4}=1\\ & r_{4}^{\prime }=r_{4}-1,r_{4}=2,3,\ldots ,S_{2}\\ \text{0}, & \text{otherwise} \end{array}\right.$$

$$Y_{r_{2}}^{\prime \prime \prime }=\left\{ \begin{array}{cc} p_{1}\mu_{1}^{\prime }, & r_{5}^{\prime }=r_{5},r_{6}^{\prime }=r_{6}-1;r_{5}=0,r_{6}=1,2,\ldots ,N\\ & r_{5}^{\prime }=r_{5}-1,r_{6}^{\prime }=r_{6}-1;r_{5}=1,r_{6}=1,2,\ldots ,N\\ 0, & \text{otherwise} \end{array}\right.$$

$$Y_{r_{2}}^{\prime \prime }=\left\{ \begin{array}{cc} Y_{r_{2}}^{⁗}, & r_{4}^{\prime }=r_{4},r_{4}=1,2,\ldots ,S_{2}\\ \text{0}, & \text{otherwise} \end{array}\right.$$

$$Y_{r_{2}}^{⁗}=\left\{ \begin{array}{cc} \gamma_{1}, & r_{5}^{\prime }=r_{5},r_{6}^{\prime }=r_{6};r_{5}=0,r_{6}=0\\ & r_{5}^{\prime }=r_{5},r_{6}^{\prime }=r_{6};r_{5}=0,1,r_{6}=1,2,\ldots ,N\\ 0, & \text{otherwise} \end{array}\right..$$

For $r_{2}=s+1$,

$$Y_{r_{2}}=\left\{ \begin{array}{cc} Y_{r_{2}}^{\prime }, & r_{3}^{\prime }=r_{3},r_{3}=0,1,2,\ldots ,S_{1}-(s+1)\\ Y_{r_{2}}^{\prime \prime } & r_{3}^{\prime }=0,r_{3}=0,1,2,\ldots ,S_{1}-(s+1)\\ \text{0}, & \text{otherwise} \end{array}\right.$$

$$Y_{r_{2}}^{\prime }=\left\{ \begin{array}{cc} Y_{r_{3}}^{\prime \prime \prime }, & r_{4}^{\prime }=S_{2},r_{4}=1\\ & r_{4}^{\prime }=r_{4}-1,r_{4}=2,3,\ldots ,S_{2}\\ \text{0}, & \text{otherwise} \end{array}\right.$$

$$Y_{r_{2}}^{\prime \prime \prime }=\left\{ \begin{array}{cc} p_{1}\mu_{1}^{\prime }, & r_{5}^{\prime }=r_{5},r_{6}^{\prime }=r_{6}-1;r_{5}=0,r_{6}=1,2,\ldots ,n+1\\ & r_{5}^{\prime }=r_{5}+1,r_{6}^{\prime }=r_{6}-1;r_{5}=0,r_{6}=n+2,n+3,\ldots ,N\\ p_{1}\mu_{1}, & r_{5}^{\prime }=r_{5}-1,r_{6}^{\prime }=r_{6}-1;r_{5}=1,r_{6}=1\\ & r_{5}^{\prime }=r_{5},r_{6}^{\prime }=r_{6}-1;r_{5}=1,r_{6}=2,3,\ldots ,N\\ 0, & \text{otherwise} \end{array}\right.$$

$$Y_{r_{2}}^{\prime \prime }=\left\{ \begin{array}{cc} Y_{r_{2}}^{⁗}, & r_{4}^{\prime }=r_{4},r_{4}=1,2,\ldots ,S_{2}\\ \text{0}, & \text{otherwise} \end{array}\right.$$

$$Y_{r_{2}}^{⁗}=\left\{ \begin{array}{cc} r_{2}\gamma_{1}, & r_{5}^{\prime }=r_{5},r_{6}^{\prime }=r_{6};r_{5}=0,r_{6}=0\\ & r_{5}^{\prime }=r_{5},r_{6}^{\prime }=r_{6};r_{5}=0,1,r_{6}=1,2,\ldots ,N\\ 0, & \text{otherwise} \end{array}\right..$$

For $r_{2}\neq 1,s+1$,

$$Y_{r_{2}}=\left\{ \begin{array}{cc} Y_{r_{2}}^{\prime }, & r_{3}^{\prime }=r_{3},r_{3}=0,1,2,\ldots ,s-r_{2}\;\;\;\;\;\;\;\;\;\;\text{if}\;r_{2}\in G_{0}^{s}\\ & r_{3}^{\prime }=r_{3},r_{3}=0,1,2,\ldots ,S_{1}-r_{2}\;\;\;\;\;\;\;\text{ if}\;r_{2}\in G_{s+1}^{S_{1}}\\ Y_{r_{2}}^{\prime \prime }, & r_{3}^{\prime }=r_{3}+1,r_{3}=0,1,2,\ldots ,s-r_{2}\;\;\;\;\text{if}\;r_{2}\in G_{0}^{s}\\ & r_{3}^{\prime }=r_{3}+1,r_{3}=0,1,2,\ldots ,S_{1}-r_{2}\;\;\text{if}\;r_{2}\in G_{s+1}^{S_{1}}\\ \text{0}, & \text{otherwise} \end{array}\right.$$

$$Y_{r_{2}}^{\prime }=\left\{ \begin{array}{cc} Y_{r_{3}}^{\prime \prime \prime }, & r_{4}^{\prime }=S_{2},r_{4}=1\\ & r_{4}^{\prime }=r_{4}-1,r_{4}=2,3,\ldots ,S_{2}\\ \text{0}, & \text{otherwise} \end{array}\right.$$

$$Y_{r_{2}}^{\prime \prime \prime }=\left\{ \begin{array}{cc} p_{1}\mu_{1}^{\prime }, & r_{5}^{\prime }=r_{5},r_{6}^{\prime }=r_{6}-1;r_{5}=0,r_{6}=1,2\ldots ,n+1\\ & r_{5}^{\prime }=r_{5}+1,r_{6}^{\prime }=r_{6}-1;r_{5}=0,r_{6}=n+2,n+3,\ldots ,N\\ p_{1}\mu_{1}, & r_{5}^{\prime }=r_{5}-1,r_{6}^{\prime }=r_{6}-1;r_{5}=1,r_{6}=1\\ & r_{5}^{\prime }=r_{5},r_{6}^{\prime }=r_{6}-1;r_{5}=1,r_{6}=2,3,\ldots ,N\\ 0, & \text{Otherwise} \end{array}\right.$$

$$Y_{r_{2}}^{\prime \prime }=\left\{ \begin{array}{cc} Y_{r_{2}}^{⁗}, & r_{4}^{\prime }=r_{4},r_{4}=1,2,\ldots ,S_{2}\\ \text{0}, & \text{otherwise} \end{array}\right.$$

$$Y_{r_{2}}^{⁗}=\left\{ \begin{array}{cc} r_{2}\gamma_{1}, & r_{5}^{\prime }=r_{5},r_{6}^{\prime }=r_{6};r_{5}=0,r_{6}=0\\ & r_{5}^{\prime }=r_{5},r_{6}^{\prime }=r_{6};r_{5}=0,1,r_{6}=1,2,\ldots ,N\\ 0, & \text{otherwise} \end{array}\right..$$

For $r_{2}=0,1,2,\ldots ,S_{1}$,

$$X_{r_{1},r_{2}}=\left\{ \begin{array}{cc} X_{r_{1},r_{2}}^{\prime }, & r_{3}^{\prime }=r_{3},r_{3}=0,1,2,\ldots ,s-r_{2}\;\;\;\;\text{if}\;r_{2}\in G_{0}^{s}\\ & r_{3}^{\prime }=r_{3},r_{3}=0,1,2,\ldots ,S_{1}-r_{2}\;\;\text{if}\;r_{2}\in G_{s+1}^{S_{1}}\\ \text{0}, & \text{otherwise} \end{array}\right.$$

$$X_{r_{1},r_{2}}^{\prime }=\left\{ \begin{array}{cc} X_{r_{1},r_{2},r_{4}}^{\prime \prime }, & r_{4}^{\prime }=r_{4},r_{4}=1,2,\ldots ,S_{2}\\ X_{r_{1},r_{2},r_{4}}^{\prime \prime \prime }, & r_{4}^{\prime }=S_{2},r_{4}=1\\ & r_{4}^{\prime }=r_{4}-1,r_{4}=2,3,\ldots ,S_{2}\\ \text{0}\;, & \text{otherwise} \end{array}\right..$$

For $r_{2}=0$,

$$X_{r_{1},r_{2},r_{4}}^{\prime \prime }=\left\{ \begin{array}{cc} \lambda_{0}, & r_{5}^{\prime }=r_{5},r_{6}^{\prime }=r_{6}+1;r_{5}=0,r_{6}=0,1,2,\ldots N-1\\ p_{3}\mu_{3}^{\prime }, & r_{5}^{\prime }=r_{5},r_{6}^{\prime }=r_{6}-1;r_{5}=0,r_{6}=1,2,\ldots N\\ -(\beta +\lambda_{0}{\overset{¯}{\delta }}_{r_{6}N}+r\lambda_{0}\delta_{r_{6}N}+ & r_{5}^{\prime }=r_{5},r_{6}^{\prime }=r_{6};r_{5}=0,r_{6}=0,1,2,\ldots ,N\\ r_{1}\theta_{0}{\overset{¯}{\delta }}_{r_{6}N}{\overset{¯}{\delta }}_{r_{1}0}+r_{4}\gamma_{2}+ & \\ (p_{2}\mu_{2}^{\prime }+p_{3}\mu_{3}^{\prime }+p_{4}\mu_{3}^{\prime })\delta_{r_{1}0}), & \\ 0, & \text{otherwise} \end{array}\right.$$

$$X_{r_{1},r_{2},r_{4}}^{\prime \prime \prime }=\left\{ \begin{array}{cc} r_{4}\gamma_{2}, & r_{5}^{\prime }=r_{5},r_{6}^{\prime }=r_{6};r_{5}=0,r_{6}=0,1,2,\ldots ,N\\ p_{2}\mu_{2}^{\prime }, & r_{5}^{\prime }=r_{5},r_{6}^{\prime }=r_{6}-1;r_{5}=0,r_{6}=1,2,\ldots ,N\\ 0, & \text{otherwise} \end{array}\right..$$

For $r_{2}=1,2,\ldots ,S_{1}$,

$$X_{r_{1},r_{2},r_{4}}^{\prime \prime }=\left\{ \begin{array}{cc} \lambda_{j}, & r_{5}^{\prime }=r_{5},r_{6}^{\prime }=r_{6}+1;r_{5}=0,r_{6}=0\\ & r_{5}^{\prime }=r_{5},r_{6}^{\prime }=r_{6}+1;r_{5}=0,1,r_{6}=1,2,\ldots N-1\\ q\eta , & r_{5}^{\prime }=r_{5}+1,r_{6}^{\prime }=r_{6};r_{5}=0,r_{6}=1,2,\ldots ,n\\ p_{1}\mu_{1}^{\prime }, & r_{5}^{\prime }=r_{5},r_{6}^{\prime }=r_{6}-1;r_{5}=0,r_{6}=1,2,\ldots ,n+1\\ & r_{5}^{\prime }=r_{5}+1,r_{6}^{\prime }=r_{6}-1;r_{5}=0,r_{6}=n+2,n+3,\ldots ,N\\ p_{1}\mu_{1}, & r_{5}^{\prime }=r_{5}-1,r_{6}^{\prime }=r_{6}-1;r_{5}=1,r_{6}=1\\ & r_{5}^{\prime }=r_{5},r_{5}=1;r_{6}^{\prime }=r_{6}-1,r_{6}=2,3,\ldots ,N\\ -(\lambda_{j}{\overset{¯}{\delta }}_{r_{6}N}+r\lambda_{j}\delta_{r_{6}N}+ & r_{5}^{\prime }=r_{5},r_{6}^{\prime }=r_{6};r_{5}=0,r_{6}=0,1,2,\ldots ,N\\ (\sum_{k=1}^{3}p_{k}\mu_{k}+p_{4}\mu_{3})\delta_{r_{5}1}+ & \\ (p\sum_{k=1}^{3}p_{k}\mu_{k}^{\prime }+p_{4}\mu_{3}^{\prime })\delta_{r_{5}0}{\overset{¯}{\delta }}_{r_{6}0}+ & \\ r_{1}\theta_{j}{\overset{¯}{\delta }}_{r_{6}N}{\overset{¯}{\delta }}_{r_{1}0}+\beta H(s-r_{2})+\\ r_{2}\gamma_{1}+r_{4}\gamma_{2}+q\eta \delta_{r_{5}0}{\overset{¯}{\delta }}_{r_{6}0}), & \\ 0, & \text{otherwise} \end{array}\right.$$

where j=1,2,…,c and $r_{2}\in I_{j}$.

$$X_{r_{1},r_{2},r_{4}}^{\prime \prime \prime }=\left\{ \begin{array}{cc} r_{4}\gamma_{2}, & r_{5}^{\prime }=r_{5},r_{6}^{\prime }=r_{6};r_{5}=0,r_{6}=0\\ & r_{5}^{\prime }=r_{5},r_{6}^{\prime }=r_{6};r_{5}=0,1,r_{6}=1,2,\ldots ,N\\ p_{2}\mu_{2}^{\prime }, & r_{5}^{\prime }=r_{5},r_{6}^{\prime }=r_{6}-1;r_{5}=0,r_{6}=1,2,\ldots ,n+1\\ & r_{5}^{\prime }=r_{5}+1,r_{6}^{\prime }=r_{6}-1;r_{5}=0,r_{6}=n+2,n+3,\ldots ,N\\ p_{2}\mu_{2}, & r_{5}^{\prime }=r_{5}-1,r_{6}^{\prime }=r_{6}-1;r_{5}=1,r_{6}=1\\ & r_{5}^{\prime }=r_{5},r_{6}^{\prime }=r_{6}-1;r_{5}=1,r_{6}=2,3,\ldots ,N\\ 0, & \text{otherwise} \end{array}\right.$$

### 3.2 Stability analysis of the system

As the considered QBD process is level-dependent, in order to compute the steady-state probabilities, Neuts and Rao's truncation method [46] is applied. So that the infinitesimal generator matrix *P* in (1) is truncated at the cut-off point *ℓ*. Then, the new modified infinitesimal generator matrix $P^{⁎}$ is given by

(2) $$P^{⁎}=\left(\begin{array}{ccccccc} P_{0,0} & P_{0,1} & & & & & \\ P_{1,0} & P_{1,1} & P_{0,1} & & & & \\ & P_{2,0} & P_{2,2} & P_{0,1} & & & \\ & & \ddots & \ddots & \ddots & & \\ & & & P_{\ell ,0} & P_{\ell ,\ell } & P_{0,1}\\ & & & & P_{\ell ,0} & P_{\ell ,\ell } & P_{0,1}\\ & & & & \ddots & \ddots & \ddots \end{array}\right).$$

Let $\phi =(\phi^{\left(0\right)},\phi^{\left(1\right)},\phi^{\left(2\right)},\ldots ,\phi^{\left(S_{1}\right)})$ be the probability of the generator $P_{\ell }=P_{\ell ,0}+P_{\ell ,\ell }+P_{0,1}$, where

$$P_{\ell }=\left\{ \begin{array}{cc} U_{r_{2}}, & r_{2}^{\prime }=r_{2},r_{2}=0,1,\ldots S_{1}\\ Y_{r_{2}}, & r_{2}^{\prime }=r_{2}-1,r_{2}=1,2,\ldots S_{1}\\ Z_{r_{2}}, & r_{2}^{\prime }=r_{2}+Q,r_{2}=0,1,\ldots s\\ \text{0}, & \text{otherwise} \end{array}\right.$$

$$U_{r_{2}}=\left\{ \begin{array}{cc} U_{r_{2}}^{\prime }, & r_{3}^{\prime }=r_{3},r_{3}=0,1,2,\ldots ,s-r_{2}\;\;\;\;\text{if}\;r_{2}\in G_{0}^{s}\\ & r_{3}^{\prime }=r_{3},r_{3}=0,1,2,\ldots ,S_{1}-r_{2}\;\;\text{if}\;r_{2}\in G_{s+1}^{S_{1}}\\ \text{0}, & \text{otherwise} \end{array}\right.$$

$$U_{r_{2}}^{\prime }=\left\{ \begin{array}{cc} U_{r_{2},r_{4}}^{\prime \prime }, & r_{4}^{\prime }=r_{4},r_{4}=1,2,\ldots ,S_{2}\\ X_{\ell ,r_{2},r_{4}}^{\prime \prime \prime }, & r_{4}^{\prime }=S_{2},r_{4}=1\\ & r_{4}^{\prime }=r_{4}-1,r_{4}=2,3,\ldots ,S_{2}\\ \text{0}, & \text{otherwise} \end{array}\right.$$

For $r_{2}=0$,

$$U_{r_{2},r_{4}}^{\prime \prime }=\left\{ \begin{array}{cc} \lambda_{0}+\ell \theta_{0}, & r_{5}^{\prime }=r_{5},r_{6}^{\prime }=r_{6}+1;r_{5}=0,r_{6}=0,1,2,\ldots N-1\\ (p_{3}+p_{4})\mu_{3}^{\prime }, & r_{5}^{\prime }=r_{5},r_{6}^{\prime }=r_{6}-1;r_{5}=0,r_{6}=1,2,\ldots N\\ -(\beta +(\lambda_{0}+\ell \theta_{0}){\overset{¯}{\delta }}_{r_{6}N}+r_{4}\gamma_{2} & r_{5}^{\prime }=r_{5},r_{6}^{\prime }=r_{6};r_{5}=0,r_{6}=0,1,2,\ldots ,N\\ +(p_{2}\mu_{2}^{\prime }+p_{3}\mu_{3}^{\prime }+p_{4}\mu_{3}^{\prime }){\overset{¯}{\delta }}_{r_{6}0}), & \\ 0, & \text{otherwise} \end{array}\right.$$

For $r_{2}=1,2,\ldots ,S_{1}$

$$U_{r_{2},r_{4}}^{\prime \prime }=\left\{ \begin{array}{cc} \lambda_{j}+\ell \theta_{j}, & r_{5}^{\prime }=r_{5},r_{6}^{\prime }=r_{6}+1;r_{5}=0,r_{6}=0\\ & r_{5}^{\prime }=r_{5},r_{6}^{\prime }=r_{6}+1;r_{5}=0,1,r_{6}=1,2,\ldots N-1\\ q\eta , & r_{5}^{\prime }=r_{5}+1,r_{6}^{\prime }=r_{6};r_{5}=0,r_{6}=1,2,\ldots ,n\\ (p_{3}+p_{4})\mu_{3}^{\prime }, & r_{5}^{\prime }=r_{5},r_{6}^{\prime }=r_{6}-1;r_{5}=0,r_{6}=1,2,\ldots ,n+1\\ & r_{5}^{\prime }=r_{5}+1,r_{6}^{\prime }=r_{6}-1;r_{5}=0,r_{6}=n+2,n+3,\ldots ,N\\ (p_{3}+p_{4})\mu_{3}, & r_{5}^{\prime }=r_{5}-1,r_{6}^{\prime }=r_{6}-1;r_{5}=1,r_{6}=1\\ & r_{5}^{\prime }=r_{5},r_{6}^{\prime }=r_{6}-1;r_{5}=1,r_{6}=2,3,\ldots ,N\\ -((\lambda_{j}+\ell \theta_{j}){\overset{¯}{\delta }}_{r_{6}N}+r_{2}\gamma_{1}+ & r_{5}^{\prime }=r_{5},r_{6}^{\prime }=r_{6};r_{5}=0,r_{6}=0,1,2,\ldots ,N\\ (\sum_{k=1}^{3}p_{k}\mu_{k}+p_{4}\mu_{3})\delta_{r_{5}1}+r_{4}\gamma_{2}+ & \\ (\sum_{k=1}^{3}p_{k}\mu_{k}^{\prime }+p_{4}\mu_{3}^{\prime })\delta_{r_{5}0}{\overset{¯}{\delta }}_{r_{6}0}+ & \\ \beta H(s-r_{2})+q\eta \delta_{r_{5}0}{\overset{¯}{\delta }}_{r_{6}0}), & \\ 0, & \text{otherwise} \end{array}\right.$$

where j=1,2,…,c and $r_{2}\in I_{j}$.

Theorem 1 *The steady state probability vector* $\phi =(\phi^{\left(0\right)},\;\phi^{\left(1\right)},\;\phi^{\left(2\right)},\ldots ,\;\phi^{\left(S_{1}\right)})$ *to the matrix* $P_{\ell }$ *is given by*

(3) $$\phi^{\left(r_{2}\right)}=\phi^{\left(Q\right)}\chi_{r_{2}}\;\forall \;r_{2}=0,1,2,\ldots ,S_{1}$$

*where*

$$\chi_{r_{2}}=\left\{ \begin{array}{cc} -\chi_{r_{2}+1}Y_{r_{2}+1}U_{r_{2}}^{-1}, & \;\text{for}\;r_{2}=0,1,2,\ldots ,Q-2\\ -Y_{r_{2}+1}U_{r_{2}}^{-1}, & \;\text{for}\;r_{2}=Q-1\\ I, & \;\text{for}\;r_{2}=Q\\ -(\chi_{r_{2}-Q}Z_{r_{2}-Q}+\chi_{r_{2}+1}Y_{r_{2}+1})U_{r_{2}}^{-1}, & \;\text{for}\;r_{2}=Q+1,Q+2,\ldots ,S_{1}-1\\ -\chi_{s}Z_{s}U_{S_{1}}^{-1}, & \;\text{for}\;r_{2}=S_{1} \end{array}\right.$$

Proof Let $\phi =(\phi^{\left(0\right)},\;\phi^{\left(1\right)},\;\phi^{\left(2\right)},\ldots ,\;\phi^{\left(S_{1}\right)})$ be the steady state probability vector which satisfies

(4) $$\phi P_{\ell }=\text{0}\;\text{ and }\phi \text{e}=1.$$

Writing $\phi P_{\ell }=\text{0}$ from (4) explicitly, we get the following set of equations

(5) $$\phi^{\left(r_{2}\right)}U_{r_{2}}+\phi^{\left(r_{2}+1\right)}Y_{r_{2}+1}=\text{0}r_{2}=0,1,2,\ldots ,Q-1$$

(6) $$\phi^{\left(r_{2}-Q\right)}Z_{r_{2}-Q}+\phi^{\left(r_{2}\right)}U_{r_{2}}+\phi^{\left(r_{2}+1\right)}Y_{r_{2}+1}=\text{0}r_{2}=Q,Q+1,Q+2,\ldots ,S_{1}-1$$

(7) $$\phi^{\left(r_{2}-Q\right)}Z_{r_{2}-Q}+\phi^{\left(r_{2}\right)}U_{r_{2}}=\text{0}r_{2}=S_{1}.$$

Consider $\phi^{\left(Q-1\right)}U_{Q-1}+\phi^{\left(Q\right)}Y_{Q}=\text{0}$ from (5), then

$$\phi^{\left(Q-1\right)}=-\phi^{\left(Q\right)}\chi_{Q-1}\;\;\text{where}\;\;\chi_{Q-1}=-Y_{Q}U_{Q-1}^{-1}$$

Consider $\phi^{\left(Q-2\right)}U_{Q-2}+\phi^{\left(Q-1\right)}Y_{Q-1}=\text{0}$ from (5), then

$$\phi^{\left(Q-2\right)}=-\phi^{\left(Q\right)}\chi_{Q-2}\;\;\text{where}\;\;\chi_{Q-2}=-\chi_{Q-1}Y_{Q-1}U_{Q-2}^{-1}.$$

Similarly solving (5) recursively in the backward, we get

(8) $$\phi^{\left(r_{2}\right)}=\phi^{\left(Q\right)}\chi_{r_{2}}\;\forall \;r_{2}=0,1,2,\ldots ,Q$$

where $\chi_{r_{2}}=\left\{ \begin{array}{cc} -\chi_{r_{2}+1}Y_{r_{2}+1}U_{r_{2}}^{-1}, & r_{2}=0,1,2,\ldots ,Q-2\\ -Y_{Q}U_{Q-1}^{-1}, & r_{2}=Q-1\\ I, & r_{2}=Q \end{array}\right.$.

From (7), we get

(9) $$\phi^{\left(S_{1}\right)}=\phi^{\left(Q\right)}\chi_{S_{1}}\;\;\text{where}\;\;\chi_{S_{1}}=-\chi_{s}Z_{s}U_{S_{1}}^{-1}.$$

Similarly solving (6) recursively in backward, we get

(10) $$\phi^{\left(r_{2}\right)}=\phi^{\left(Q\right)}\chi_{r_{2}}\;\forall \;r_{2}=Q+1,Q+2,\ldots ,S_{1}-1,$$

where $\chi_{r_{2}}=-(\chi_{r_{2}-Q}Z_{r_{2}-Q}+\chi_{r_{2}+1}Y_{r_{2}+1})U_{r_{2}}^{-1}\;\;\forall \;\;r_{2}=Q+1,Q+2,\ldots ,S_{1}-1$.

Combining equations (8), (9) and (10), we get (3). Also, solving $\phi^{\left(Q\right)}(-\chi_{1}Y_{1}U_{0}^{-1}Z_{0}+I-(\chi_{1}Z_{1}+\chi_{2}Y_{2})U_{Q+1}^{-1}Y_{Q+1})=\text{0}$ and using the normalizing condition $\sum_{r_{2}=0}^{S_{1}}\phi^{\left(r_{2}\right)}=1$, we obtain the steady-state probability vector $\phi^{\left(Q\right)}$.

Theorem 2 *The considered queuing-inventory system is stable under the condition of*

(11) $$f_{1}>f_{2}$$

*where*

$$f_{1}=\sum_{r_{3}=0}^{s}\sum_{r_{4}=1}^{S_{2}}\sum_{r_{6}=0}^{N-1}\ell \theta_{0}\phi^{\left(0,r_{3},r_{4},0,r_{6}\right)}+\sum_{r_{2}=1}^{s}\sum_{r_{3}=0}^{s-r_{2}}\sum_{r_{4}=1}^{S_{2}}\sum_{r_{6}=0}^{N-1}\ell \theta_{j}\phi^{\left(r_{2},r_{3},r_{4},0,r_{6}\right)}+\;\;\;\;\;\;\;\;\;\sum_{r_{2}=1}^{s}\sum_{r_{3}=0}^{s-r_{2}}\sum_{r_{4}=1}^{S_{2}}\sum_{r_{6}=1}^{N-1}\ell \theta_{j}\phi^{\left(r_{2},r_{3},r_{4},1,r_{6}\right)}+\sum_{r_{2}=s+1}^{S_{1}}\sum_{r_{3}=0}^{S_{1}-r_{2}}\sum_{r_{4}=1}^{S_{2}}\sum_{r_{6}=0}^{N-1}\ell \theta_{j}\phi^{\left(r_{2},r_{3},r_{4},0,r_{6}\right)}+\;\;\;\;\;\;\;\;\sum_{r_{2}=s+1}^{S_{1}}\sum_{r_{3}=0}^{S_{1}-r_{2}}\sum_{r_{4}=1}^{S_{2}}\sum_{r_{6}=1}^{N-1}\ell \theta_{j}\phi^{\left(r_{2},r_{3},r_{4},1,r_{6}\right)}\;\;\text{and}\;f_{2}=\sum_{r_{3}=0}^{s}\sum_{r_{4}=1}^{S_{2}}[\sum_{r_{6}=1}^{N}p_{4}\mu_{3}^{\prime }\phi^{\left(0,r_{3},r_{4},0,r_{6}\right)}+r\lambda_{0}\phi^{\left(0,r_{3},r_{4},0,N\right)}]+\;\;\;\;\;\;\;\;\;\;\sum_{r_{2}=1}^{s}\sum_{r_{3}=0}^{s-r_{2}}\sum_{r_{4}=1}^{S_{2}}[\sum_{r_{5}=0,1}r\lambda_{j}\phi^{\left(r_{2},r_{3},r_{4},r_{5},r_{6}\right)}+\sum_{r_{6}=1}^{N}p_{4}\mu_{3}^{\prime }\phi^{\left(r_{2},r_{3},r_{4},0,r_{6}\right)}+\sum_{r_{6}=1}^{N}p_{4}\mu_{3}\phi^{\left(r_{2},r_{3},r_{4},1,r_{6}\right)}]+\;\;\;\;\;\;\;\;\;\;\sum_{r_{2}=s+1}^{S_{1}}\sum_{r_{3}=0}^{S_{1}-r_{2}}\sum_{r_{4}=1}^{S_{2}}[\sum_{r_{5}=0,1}r\lambda_{j}\phi^{\left(r_{2},r_{3},r_{4},r_{5},N\right)}+\sum_{r_{6}=1}^{N}p_{4}\mu_{3}^{\prime }\phi^{\left(r_{2},r_{3},r_{4},0,r_{6}\right)}+\sum_{r_{6}=1}^{N}p_{4}\mu_{3}\phi^{\left(r_{2},r_{3},r_{4},1,r_{6}\right)}].$$

Proof Using the condition from Neuts [47], $\phi P_{\ell ,0}\text{e}>\phi P_{0,1}\text{e}$, the stability condition for the system can be obtained.

Let us consider

$$\phi_{\ell ,0}\text{e}=(\sum_{r_{2}=0}^{S_{1}}\phi^{\left(r_{2}\right)}W_{r_{2}})\text{e}=(\sum_{r_{2}=0}^{s}\sum_{r_{3}=0}^{s-r_{2}}\phi^{\left(r_{2},r_{3}\right)}W_{r_{2}}^{\prime }+\sum_{r_{2}=s+1}^{S_{1}}\sum_{r_{3}=0}^{S_{1}-r_{2}}\phi^{\left(r_{2},r_{3}\right)}W_{r_{2}}^{\prime })\text{e}=(\sum_{r_{2}=0}^{s}\sum_{r_{3}=0}^{s-r_{2}}\sum_{r_{4}=1}^{S_{2}}\phi^{\left(r_{2},r_{3},r_{4}\right)}W_{r_{2}}^{\prime \prime }+\sum_{r_{2}=s+1}^{S_{1}}\sum_{r_{3}=0}^{S_{1}-r_{2}}\sum_{r_{4}=1}^{S_{2}}\phi^{\left(r_{2},r_{3},r_{4}\right)}W_{r_{2}}^{\prime \prime })\text{e}$$

Expanding the above, we get $f_{1}$.

Let us consider

$$\phi P_{0,1}\text{e}=(\sum_{r_{2}=0}^{S_{1}}\phi^{\left(r_{2}\right)}V_{r_{2}})\text{e}=(\sum_{r_{2}=0}^{s}\sum_{r_{3}=0}^{s-r_{2}}\phi^{\left(r_{2},r_{3}\right)}V_{r_{2}}^{\prime }+\sum_{r_{2}=s+1}^{S_{1}}\sum_{r_{3}=0}^{S_{1}-r_{2}}\phi^{\left(r_{2},r_{3}\right)}V_{r_{2}}^{\prime })\text{e}=(\sum_{r_{2}=0}^{s}\sum_{r_{3}=0}^{s-r_{2}}\sum_{r_{4}=1}^{S_{2}}\phi^{\left(r_{2},r_{3},r_{4}\right)}V_{r_{2}}^{\prime \prime }+\sum_{r_{2}=s+1}^{S_{1}}\sum_{r_{3}=0}^{S_{1}-r_{2}}\sum_{r_{4}=1}^{S_{2}}\phi^{\left(r_{2},r_{3},r_{4}\right)}V_{r_{2}}^{\prime \prime })\text{e}$$

Expanding the above, we get $f_{2}$.

Substituting $f_{1}$ and $f_{2}$ in $\phi^{\left(\ell \right)}P_{\ell ,0}\text{e}>\phi^{\left(\ell \right)}P_{0,1}\text{e}$, we yield (11).

## 4 Computation of steady-state probability vector

In this section, the steady state probability vector is derived by using the rate matrix R.

The structural pattern of $P^{⁎}$ shows the regularity of the continuous-time Markov chain {R(t):t≥0} with state space E. As a result, the rate matrix $P^{⁎}$ has a limiting distribution $\Pi =(\Pi^{\left(0\right)},\Pi^{\left(1\right)},\Pi^{\left(2\right)},\ldots )$ that is independent of the initial state which is given by

$$\Pi^{\left(r_{1},r_{2},r_{3},r_{4},r_{5},r_{6}\right)}=\lim_{t\to \infty }Pr\{(R_{1}(t)=r_{1},R_{2}(t)=r_{2},R_{3}(t)=r_{3},R_{4}(t)=r_{4},R_{5}(t)=r_{5},\;\;\;\;\;\;R_{6}(t)=r_{6})|(R_{1}(0),R_{2}(0),R_{3}(0),R_{4}(0),R_{5}(0),R_{6}(0))\}.$$

According to the following manner the limiting probability vector is divided into sub-vectors.

$$\Pi^{\left(r_{1}\right)}=(\Pi^{\left(r_{1},0\right)},\;\Pi^{\left(r_{1},1\right)},\;\Pi^{\left(r_{1},2\right)},\ldots ,\Pi^{\left(r_{1},S_{1}\right)})\Pi^{\left(r_{1},r_{2}\right)}=\left\{ \begin{array}{cc} (\Pi^{\left(r_{1},r_{2},0\right)},\Pi^{\left(r_{1},r_{2},1\right)},\ldots ,\Pi^{\left(r_{1},r_{2},s-r_{3}\right)})\; & \;\forall \;r_{3}=0,1,2,\ldots s\\ (\Pi^{\left(r_{1},r_{2},0\right)},\Pi^{\left(r_{1},r_{2},1\right)},\ldots ,\Pi^{\left(r_{1},r_{2},S_{1}-r_{3}\right)})\; & \;\forall \;r_{3}=s+1,s+2,\ldots ,S_{1} \end{array}\right.\;\Pi^{\left(r_{1},r_{2},r_{3}\right)}=(\Pi^{\left(r_{1},r_{2},r_{3},1\right)},\Pi^{\left(r_{1},r_{2},r_{3},2\right)},\ldots ,\Pi^{\left(r_{1},r_{2},r_{3},S_{2}\right)})\Pi^{\left(r_{1},r_{2},r_{3},r_{4}\right)}=\left\{ \begin{array}{cc} \left(\Pi^{\left(r_{1},r_{2},r_{3},r_{4},0,0\right)},\Pi^{\left(r_{1},r_{2},r_{3},r_{4},0,1\right)},\ldots ,\Pi^{\left(r_{1},r_{2},r_{3},r_{4},0,N\right)}\right) & \text{for }r_{2}=0,1,2,\ldots ,S_{1}\\ \left(\Pi^{\left(r_{1},r_{2},r_{3},r_{4},1,1\right)},\Pi^{\left(r_{1},r_{2},r_{3},r_{4},1,2\right)},\ldots ,\Pi^{\left(r_{1},r_{2},r_{3},r_{4},1,N\right)}\right) & \text{for }r_{2}=1,2,\ldots ,S_{1} \end{array}\right.$$

Then the limiting distribution satisfies $\Pi P^{⁎}=0$ and Πe=1.

### 4.1 Computation of R matrix

In order to find the steady-state probability vector $\Pi =(\Pi^{\left(0\right)},\Pi^{\left(1\right)},\Pi^{\left(2\right)},\ldots )$ to the infinitesimal generator matrix $P^{⁎}$ in (2), it is necessary to compute the rate matrix *R*. The rate matrix *R* is the minimal non-negative solution of the matrix quadratic equation

(12) $$P_{\ell ,0}R^{2}+P_{\ell ,\ell }R+P_{0,1}=\text{0}.$$

The entries of the rate matrix are given by

$$R=\left(\begin{array}{ccccc} R_{\left(0\right)}^{\left(0\right)} & R_{\left(1\right)}^{\left(0\right)} & R_{\left(2\right)}^{\left(0\right)} & \ldots & R_{\left(S_{1}\right)}^{\left(0\right)}\\ R_{\left(0\right)}^{\left(1\right)} & R_{\left(1\right)}^{\left(1\right)} & R_{\left(2\right)}^{\left(1\right)} & \ldots & R_{\left(S_{1}\right)}^{\left(1\right)}\\ \vdots & \vdots & \vdots & & \vdots \\ R_{\left(0\right)}^{\left(S_{1}\right)} & R_{\left(1\right)}^{\left(S_{1}\right)} & R_{\left(2\right)}^{\left(S_{1}\right)} & \ldots & R_{\left(S_{1}\right)}^{\left(S_{1}\right)} \end{array}\right).$$

For $r_{2}\in G_{0}^{s}$ and $r_{2}^{\prime }\in G_{0}^{s}$,

$$R_{\left(r_{2}^{\prime }\right)}^{\left(r_{2}\right)}=\left(\begin{array}{ccccc} R_{\left(r_{2}^{\prime },0\right)}^{\left(r_{2},0\right)} & R_{\left(r_{2}^{\prime },1\right)}^{\left(r_{2},0\right)} & R_{\left(r_{2}^{\prime },2\right)}^{\left(r_{2},0\right)} & \ldots & R_{\left(r_{2}^{\prime },s-r_{2}^{\prime }\right)}^{\left(r_{2},0\right)}\\ R_{\left(r_{2}^{\prime },0\right)}^{\left(r_{2},1\right)} & R_{\left(r_{2}^{\prime },1\right)}^{\left(r_{2},1\right)} & R_{\left(r_{2}^{\prime },2\right)}^{\left(r_{2},1\right)} & \ldots & R_{\left(r_{2}^{\prime },s-r_{2}^{\prime }\right)}^{\left(r_{2},1\right)}\\ \vdots & \vdots & \vdots & & \vdots \\ R_{\left(r_{2}^{\prime },0\right)}^{\left(r_{2},s-r_{2}\right)} & R_{\left(r_{2}^{\prime },1\right)}^{\left(r_{2},s-r_{2}\right)} & R_{\left(r_{2}^{\prime },2\right)}^{\left(r_{2},s-r_{2}\right)} & \ldots & R_{\left(r_{2}^{\prime },s-r_{2}^{\prime }\right)}^{\left(r_{2},s-r_{2}\right)} \end{array}\right).$$

For $r_{2}\in G_{0}^{s}$ and $r_{2}^{\prime }\in G_{s+1}^{S_{1}}$,

$$R_{\left(r_{2}^{\prime }\right)}^{\left(r_{2}\right)}=\left(\begin{array}{ccccc} R_{\left(r_{2}^{\prime },0\right)}^{\left(r_{2},0\right)} & R_{\left(r_{2}^{\prime },1\right)}^{\left(r_{2},0\right)} & R_{\left(r_{2}^{\prime },2\right)}^{\left(r_{2},0\right)} & \ldots & R_{\left(r_{2}^{\prime },S_{1}-r_{2}^{\prime }\right)}^{\left(r_{2},0\right)}\\ R_{\left(r_{2}^{\prime },0\right)}^{\left(r_{2},1\right)} & R_{\left(r_{2}^{\prime },1\right)}^{\left(r_{2},1\right)} & R_{\left(r_{2}^{\prime },2\right)}^{\left(r_{2},1\right)} & \ldots & R_{\left(r_{2}^{\prime },S_{1}-r_{2}^{\prime }\right)}^{\left(r_{2},1\right)}\\ \vdots & \vdots & \vdots & & \vdots \\ R_{\left(r_{2}^{\prime },0\right)}^{\left(r_{2},s-r_{2}\right)} & R_{\left(r_{2}^{\prime },1\right)}^{\left(r_{2},s-r_{2}\right)} & R_{\left(r_{2}^{\prime },2\right)}^{\left(r_{2},s-r_{2}\right)} & \ldots & R_{\left(r_{2}^{\prime },S_{1}-r_{2}^{\prime }\right)}^{\left(r_{2},s-r_{2}\right)} \end{array}\right).$$

For $r_{2}\in G_{s+1}^{S_{1}}$ and $r_{2}^{\prime }\in G_{0}^{s}$,

$$R_{\left(r_{2}^{\prime }\right)}^{\left(r_{2}\right)}=\left(\begin{array}{ccccc} R_{\left(r_{2}^{\prime },0\right)}^{\left(r_{2},0\right)} & R_{\left(r_{2}^{\prime },1\right)}^{\left(r_{2},0\right)} & R_{\left(r_{2}^{\prime },2\right)}^{\left(r_{2},0\right)} & \ldots & R_{\left(r_{2}^{\prime },s-r_{2}^{\prime }\right)}^{\left(r_{2},0\right)}\\ R_{\left(r_{2}^{\prime },0\right)}^{\left(r_{2},1\right)} & R_{\left(r_{2}^{\prime },1\right)}^{\left(r_{2},1\right)} & R_{\left(r_{2}^{\prime },2\right)}^{\left(r_{2},1\right)} & \ldots & R_{\left(r_{2}^{\prime },s-r_{2}^{\prime }\right)}^{\left(r_{2},1\right)}\\ \vdots & \vdots & \vdots & & \vdots \\ R_{\left(r_{2}^{\prime },0\right)}^{\left(r_{2},S_{1}-r_{2}\right)} & R_{\left(r_{2}^{\prime },1\right)}^{\left(r_{2},S_{1}-r_{2}\right)} & R_{\left(r_{2}^{\prime },2\right)}^{\left(r_{2},S_{1}-r_{2}\right)} & \ldots & R_{\left(r_{2}^{\prime },s-r_{2}^{\prime }\right)}^{\left(r_{2},S_{1}-r_{2}\right)} \end{array}\right).$$

For $r_{2}\in G_{s+1}^{S_{1}}$ and $r_{2}^{\prime }\in G_{s+1}^{S_{1}}$

$$R_{\left(r_{2}^{\prime }\right)}^{\left(r_{2}\right)}=\left(\begin{array}{ccccc} R_{\left(r_{2}^{\prime },0\right)}^{\left(r_{2},0\right)} & R_{\left(r_{2}^{\prime },1\right)}^{\left(r_{2},0\right)} & R_{\left(r_{2}^{\prime },2\right)}^{\left(r_{2},0\right)} & \ldots & R_{\left(r_{2}^{\prime },S_{1}-r_{2}^{\prime }\right)}^{\left(r_{2},0\right)}\\ R_{\left(r_{2}^{\prime },0\right)}^{\left(r_{2},1\right)} & R_{\left(r_{2}^{\prime },1\right)}^{\left(r_{2},1\right)} & R_{\left(r_{2}^{\prime },2\right)}^{\left(r_{2},1\right)} & \ldots & R_{\left(r_{2}^{\prime },S_{1}-r_{2}^{\prime }\right)}^{\left(r_{2},1\right)}\\ \vdots & \vdots & \vdots & & \vdots \\ R_{\left(r_{2}^{\prime },0\right)}^{\left(r_{2},S_{1}-r_{2}\right)} & R_{\left(r_{2}^{\prime },1\right)}^{\left(r_{2},S_{1}-r_{2}\right)} & R_{\left(r_{2}^{\prime },2\right)}^{\left(r_{2},S_{1}-r_{2}\right)} & \ldots & R_{\left(r_{2}^{\prime },S_{1}-r_{2}^{\prime }\right)}^{\left(r_{2},S_{1}-r_{2}\right)} \end{array}\right).$$

For $r_{3},r_{3}^{\prime }\in G_{0}^{S_{1}-(s+1)}$,

$$R_{\left(r_{2}^{\prime },r_{3}^{\prime }\right)}^{\left(r_{2},r_{3}\right)}=\left(\begin{array}{cccc} R_{\left(r_{2}^{\prime },r_{3}^{\prime },1\right)}^{\left(r_{2},r_{3},1\right)} & R_{\left(r_{2}^{\prime },r_{3}^{\prime },2\right)}^{\left(r_{2},r_{3},1\right)} & \ldots & R_{\left(r_{2}^{\prime },r_{3}^{\prime },S_{2}\right)}^{\left(r_{2},r_{3},1\right)}\\ R_{\left(r_{2}^{\prime },r_{3}^{\prime },1\right)}^{\left(r_{2},r_{3},2\right)} & R_{\left(r_{2}^{\prime },r_{3}^{\prime },2\right)}^{\left(r_{2},r_{3},2\right)} & \ldots & R_{\left(r_{2}^{\prime },r_{3}^{\prime },S_{2}\right)}^{\left(r_{2},r_{3},2\right)}\\ \vdots & \vdots & \vdots & \vdots \\ R_{\left(r_{2}^{\prime },r_{3}^{\prime },1\right)}^{\left(r_{2},r_{3},S_{2}\right)} & R_{\left(r_{2}^{\prime },r_{3}^{\prime },2\right)}^{\left(r_{2},r_{3},S_{2}\right)} & \ldots & R_{\left(r_{2}^{\prime },r_{3}^{\prime },S_{2}\right)}^{\left(r_{2},r_{3},S_{2}\right)} \end{array}\right).$$

For all $r_{4}\in G_{1}^{S_{2}}$ and $r_{4}^{\prime }\in G_{1}^{S_{2}}$,

when $r_{2}=0$ and $r_{2}^{\prime }=0$,

$$R_{\left(r_{2}^{\prime },r_{3}^{\prime },r_{4}^{\prime }\right)}^{\left(r_{2},r_{3},r_{4}\right)}=\left(\begin{array}{cccc} 0 & 0 & \ldots & 0\\ r_{\left(r_{2}^{\prime },r_{3}^{\prime },r_{4}^{\prime },1\right)}^{\left(r_{2},r_{3},r_{4},2\right)} & r_{\left(r_{2}^{\prime },r_{3}^{\prime },r_{4}^{\prime },2\right)}^{\left(r_{2},r_{3},r_{4},2\right)} & \ldots & r_{\left(r_{2}^{\prime },r_{3}^{\prime },r_{4}^{\prime },N+1\right)}^{\left(r_{2},r_{3},r_{4},2\right)}\\ r_{\left(r_{2}^{\prime },r_{3}^{\prime },r_{4}^{\prime },1\right)}^{\left(r_{2},r_{3},r_{4},3\right)} & r_{\left(r_{2}^{\prime },r_{3}^{\prime },r_{4}^{\prime },2\right)}^{\left(r_{2},r_{3},r_{4},3\right)} & \ldots & r_{\left(r_{2}^{\prime },r_{3}^{\prime },r_{4}^{\prime },N+1\right)}^{\left(r_{2},r_{3},r_{4},3\right)}\\ \vdots & \vdots & \vdots & \vdots \\ r_{\left(r_{2}^{\prime },r_{3}^{\prime },r_{4}^{\prime },1\right)}^{\left(r_{2},r_{3},r_{4},N+1\right)} & r_{\left(r_{2}^{\prime },r_{3}^{\prime },r_{4}^{\prime },2\right)}^{\left(r_{2},r_{3},r_{4},N+1\right)} & \ldots & r_{\left(r_{2}^{\prime },r_{3}^{\prime },r_{4}^{\prime },N+1\right)}^{\left(r_{2},r_{3},r_{4},N+1\right)} \end{array}\right),$$

when $r_{2}=0$ and $r_{2}^{\prime }\neq 0$,

$$R_{\left(r_{2}^{\prime },r_{3}^{\prime },r_{4}^{\prime }\right)}^{\left(r_{2},r_{3},r_{4}\right)}=\left(\begin{array}{cccc} 0 & 0 & \ldots & 0\\ r_{\left(r_{2}^{\prime },r_{3}^{\prime },r_{4}^{\prime },1\right)}^{\left(r_{2},r_{3},r_{4},2\right)} & r_{\left(r_{2}^{\prime },r_{3}^{\prime },r_{4}^{\prime },2\right)}^{\left(r_{2},r_{3},r_{4},2\right)} & \ldots & r_{\left(r_{2}^{\prime },r_{3}^{\prime },r_{4}^{\prime },2N+1\right)}^{\left(r_{2},r_{3},r_{4},2\right)}\\ r_{\left(r_{2}^{\prime },r_{3}^{\prime },r_{4}^{\prime },1\right)}^{\left(r_{2},r_{3},r_{4},3\right)} & r_{\left(r_{2}^{\prime },r_{3}^{\prime },r_{4}^{\prime },2\right)}^{\left(r_{2},r_{3},r_{4},3\right)} & \ldots & r_{\left(r_{2}^{\prime },r_{3}^{\prime },r_{4}^{\prime },2N+1\right)}^{\left(r_{2},r_{3},r_{4},3\right)}\\ \vdots & \vdots & \vdots & \vdots \\ r_{\left(r_{2}^{\prime },r_{3}^{\prime },r_{4}^{\prime },1\right)}^{\left(r_{2},r_{3},r_{4},N+1\right)} & r_{\left(r_{2}^{\prime },r_{3}^{\prime },r_{4}^{\prime },2\right)}^{\left(r_{2},r_{3},r_{4},N+1\right)} & \ldots & r_{\left(r_{2}^{\prime },r_{3}^{\prime },r_{4}^{\prime },2N+1\right)}^{\left(r_{2},r_{3},r_{4},N+1\right)} \end{array}\right),$$

when $r_{2}\neq 0$ and $r_{2}^{\prime }=0$,

$$R_{\left(r_{2}^{\prime },r_{3}^{\prime },r_{4}^{\prime }\right)}^{\left(r_{2},r_{3},r_{4}\right)}=\left(\begin{array}{cccc} 0 & 0 & \ldots & 0\\ r_{\left(r_{2}^{\prime },r_{3}^{\prime },r_{4}^{\prime },1\right)}^{\left(r_{2},r_{3},r_{4},2\right)} & r_{\left(r_{2}^{\prime },r_{3}^{\prime },r_{4}^{\prime },2\right)}^{\left(r_{2},r_{3},r_{4},2\right)} & \ldots & r_{\left(r_{2}^{\prime },r_{3}^{\prime },r_{4}^{\prime },N+1\right)}^{\left(r_{2},r_{3},r_{4},2\right)}\\ r_{\left(r_{2}^{\prime },r_{3}^{\prime },r_{4}^{\prime },1\right)}^{\left(r_{2},r_{3},r_{4},3\right)} & r_{\left(r_{2}^{\prime },r_{3}^{\prime },r_{4}^{\prime },2\right)}^{\left(r_{2},r_{3},r_{4},3\right)} & \ldots & r_{\left(r_{2}^{\prime },r_{3}^{\prime },r_{4}^{\prime },N+1\right)}^{\left(r_{2},r_{3},r_{4},3\right)}\\ \vdots & \vdots & \vdots & \vdots \\ r_{\left(r_{2}^{\prime },r_{3}^{\prime },r_{4}^{\prime },1\right)}^{\left(r_{2},r_{3},r_{4},2N+1\right)} & r_{\left(r_{2}^{\prime },r_{3}^{\prime },r_{4}^{\prime },2\right)}^{\left(r_{2},r_{3},r_{4},2N+1\right)} & \ldots & r_{\left(r_{2}^{\prime },r_{3}^{\prime },r_{4}^{\prime },N+1\right)}^{\left(r_{2},r_{3},r_{4},2N+1\right)} \end{array}\right),$$

when $r_{2}\neq^{\prime }0$ and $r_{2}^{\prime }\neq 0$,

$$R_{\left(r_{2}^{\prime },r_{3}^{\prime },r_{4}^{\prime }\right)}^{\left(r_{2},r_{3},r_{4}\right)}=\left(\begin{array}{cccc} 0 & 0 & \ldots & 0\\ r_{\left(r_{2}^{\prime },r_{3}^{\prime },r_{4}^{\prime },1\right)}^{\left(r_{2},r_{3},r_{4},2\right)} & r_{\left(r_{2}^{\prime },r_{3}^{\prime },r_{4}^{\prime },2\right)}^{\left(r_{2},r_{3},r_{4},2\right)} & \ldots & r_{\left(r_{2}^{\prime },r_{3}^{\prime },r_{4}^{\prime },2N+1\right)}^{\left(r_{2},r_{3},r_{4},2\right)}\\ r_{\left(r_{2}^{\prime },r_{3}^{\prime },r_{4}^{\prime },1\right)}^{\left(r_{2},r_{3},r_{4},3\right)} & r_{\left(r_{2}^{\prime },r_{3}^{\prime },r_{4}^{\prime },2\right)}^{\left(r_{2},r_{3},r_{4},3\right)} & \ldots & r_{\left(r_{2}^{\prime },r_{3}^{\prime },r_{4}^{\prime },2N+1\right)}^{\left(r_{2},r_{3},r_{4},3\right)}\\ \vdots & \vdots & \vdots & \vdots \\ r_{\left(r_{2}^{\prime },r_{3}^{\prime },r_{4}^{\prime },1\right)}^{\left(r_{2},r_{3},r_{4},2N+1\right)} & r_{\left(r_{2}^{\prime },r_{3}^{\prime },r_{4}^{\prime },2\right)}^{\left(r_{2},r_{3},r_{4},2N+1\right)} & \ldots & r_{\left(r_{2}^{\prime },r_{3}^{\prime },r_{4}^{\prime },2N+1\right)}^{\left(r_{2},r_{3},r_{4},2N+1\right)} \end{array}\right).$$

Writing the equation (12) elaborately, the following set of equations are obtained.

For $r_{2}\in G_{0}^{S_{1}}$ and $r_{2}^{\prime }\in G_{0}^{S_{1}}$,

when $r_{2}=r_{2}^{\prime }$,

(13) $$\sum_{j=0}^{S_{1}}R_{\left(j\right)}^{\left(r_{2}\right)}R_{\left(r_{2}^{\prime }\right)}^{\left(j\right)}W_{r_{2}^{\prime }}+R_{\left(r_{2}^{\prime }\right)}^{\left(r_{2}\right)}U_{r_{2}^{\prime }}+R_{\left(r_{2}^{\prime }+1\right)}^{\left(r_{2}+1\right)}Y_{r_{2}^{\prime }+1}+V_{r_{2}}=\text{0},\;\;\;r_{2}^{\prime }=0,1,2,\ldots ,Q-1$$

(14) $$\sum_{j=0}^{S_{1}}R_{\left(j\right)}^{\left(r_{2}\right)}R_{\left(r_{2}^{\prime }\right)}^{\left(j\right)}W_{r_{2}^{\prime }}+R_{\left(r_{2}^{\prime }\right)}^{\left(r_{2}\right)}U_{r_{2}^{\prime }}+R_{\left(r_{2}^{\prime }+1\right)}^{\left(r_{2}+1\right)}Y_{r_{2}^{\prime }+1}+R_{\left(r_{2}^{\prime }-Q\right)}^{\left(r_{2}\right)}Z_{r_{2}^{\prime }-Q}+V_{r_{2}}=\text{0},\;\;\;r_{2}^{\prime }=Q,Q+1,\ldots ,S_{1}$$

when $r_{2}\neq r_{2}^{\prime }$,

(15) $$\sum_{j=0}^{S_{1}}R_{\left(j\right)}^{\left(r_{2}\right)}R_{\left(r_{2}^{\prime }\right)}^{\left(j\right)}W_{r_{2}^{\prime }}+R_{\left(r_{2}^{\prime }\right)}^{\left(r_{2}\right)}U_{r_{2}^{\prime }}+R_{\left(r_{2}^{\prime }+1\right)}^{\left(r_{2}+1\right)}Y_{r_{2}^{\prime }+1}=\text{0},\;\;\;r_{2}^{\prime }=0,1,2,\ldots ,Q-1$$

(16) $$\sum_{j=0}^{S_{1}}R_{\left(j\right)}^{\left(r_{2}\right)}R_{\left(r_{2}^{\prime }\right)}^{\left(j\right)}W_{r_{2}^{\prime }}+R_{\left(r_{2}^{\prime }\right)}^{\left(r_{2}\right)}U_{r_{2}^{\prime }}+R_{\left(r_{2}^{\prime }+1\right)}^{\left(r_{2}+1\right)}Y_{r_{2}^{\prime }+1}+R_{\left(r_{2}^{\prime }-Q\right)}^{\left(r_{2}\right)}Z_{r_{2}^{\prime }-Q}=\text{0},\;\;\;r_{2}^{\prime }=Q,Q+1,\ldots ,S_{1}.$$

Solving the above set of equations (13),(14),(15) and (16), the exact entries of rate matrix *R* can be obtained.

Theorem 3 *The stationary probability vector* $\Pi^{\left(r_{1}\right)},\;\forall \;r_{1}=0,1,2,\ldots$ *of the Markov chain can be determined by*

$$\Pi^{\left(r_{1}\right)}=\left\{ \begin{array}{cc} \Pi^{\left(0\right)}\Lambda_{r_{1}}, & \forall \;\;r_{1}=0,1,2,\cdots ,\ell \\ \Pi^{\left(0\right)}\Lambda_{\ell },R^{r_{1}-\ell } & \forall \;\;r_{1}>\ell \end{array}\right.$$

*where R is the minimal non-negative solution of the matrix quadratic equation* (12) *and*

$$\Lambda_{r_{1}}=\left\{ \begin{array}{cc} I & \;\text{if}\;r_{1}=0\\ \prod_{i=0}^{r_{1}}P_{0,1}A_{i} & \;\text{if}\;r_{1}=1,2,\cdots ,\ell \end{array}\right.$$

*where*

$$A_{j}=\left\{ \begin{array}{cc} -{\left(P_{0,1}A_{j+1}P_{j+2,0}+P_{\left(j+1),(j+1\right)}\right)}^{-1}, & \forall \;\;j=0,1,\cdots ,\ell -2\\ {\left[-(P_{\ell ,\ell }+RP_{\ell ,0})\right]}^{-1}, & \forall \;\;j=\ell -1 \end{array}\right.$$

Proof Let $\Pi =(\Pi^{\left(0\right)},\Pi^{\left(1\right)},\Pi^{\left(2\right)},\ldots )$ be a probability vector which satisfies

(17) $$\Pi P^{⁎}=\text{0}\;\;\;\;\text{and}\;\;\;\;\Pi \text{e}=1$$

Let us consider a rate matrix *R* which is the solution of the matrix quadratic equation (12) and assume that

(18) $$\Pi^{\left(r_{1}\right)}=\Pi^{\left(\ell \right)}R^{\left(r_{1}-\ell \right)}\forall \;\;\;\;\;r_{1}=\ell ,\ell +1,\cdots .$$

Solving $\Pi P^{⁎}=\text{0}$ from equation (17), we get the following system of equations

(19) $$\Pi^{\left(0\right)}P_{0,0}+\Pi^{\left(1\right)}P_{1,0}=\text{0}$$

(20) $$\Pi^{\left(r_{1}-1\right)}P_{0,1}+\Pi^{\left(r_{1}\right)}P_{r_{1},r_{1}}+\Pi^{\left(r_{1}+1\right)}P_{r_{1}+1,0}=\text{0}\;\;\forall \;\;\;r_{1}=1,2,\ldots ,\ell -1$$

(21) $$\Pi^{\left(\ell -1\right)}P_{0,1}+\Pi^{\left(\ell \right)}(P_{\ell ,\ell }+RP_{\ell ,0})=\text{0}$$

(22) $$\text{and}\;[\sum_{r_{1}=0}^{\ell -1}\Pi^{\left(r_{1}\right)}+\Pi^{\left(\ell \right)}{\left(I-R\right)}^{-1}]\text{e}=1.$$

From equation (21), we get

(23) $$\Pi^{\left(\ell \right)}=\Pi^{\left(\ell -1\right)}P_{0,1}A_{\ell -1},\text{ where}\;\;A_{\ell -1}={\left[-(P_{\ell ,\ell }+RP_{\ell ,0})\right]}^{-1}.$$

From (20), we get

$$\Pi^{\left(\ell -1\right)}=\Pi^{\left(\ell -2\right)}P_{0,1}A_{\ell -2},\text{ where }A_{\ell -2}={\left[-(P_{\left(\ell -1),(\ell -1\right)}+P_{0,1}G_{\ell -1}P_{\ell ,0})\right]}^{-1}.$$

Using again (20), we get

$$\Pi^{\left(\ell -2\right)}=\Pi^{\left(\ell -3\right)}P_{0,1}G_{\ell -3}\text{ where }A_{\ell -3}={\left[-(P_{\left(\ell -2),(\ell -2\right)}+P_{0,1}A_{\ell -2}P_{\ell ,0})\right]}^{-1}.$$

Similarly, solving the Equation (20), we get the general form

(24) $$\Pi^{\left(r_{1}\right)}=\Pi^{\left(r_{1}-1\right)}P_{0,1}A_{r_{1}-1}\;\;\forall \;\;r_{1}=1,2,\cdots ,\ell -1$$

where

$$A_{r_{1}}=\left\{ \begin{array}{cc} {\left[-(P_{\ell ,\ell }+RP_{\ell ,0})\right]}^{-1}, & r_{1}=\ell -1\\ {\left[-(P_{r_{1},r_{1}}+P_{0,0}A_{r_{1}}P_{r_{1}+1,1})\right]}^{-1}, & r_{1}=1,2,\cdots ,\ell -2 \end{array}\right.$$

Combining equations (23) and (24), we obtain

(25) $$\Pi^{\left(r_{1}\right)}=\Pi^{\left(0\right)}\Lambda_{r_{1}}\;\;\;\;\;\;\forall \;\;\;\;\;r_{1}=0,1,2,\cdots ,\ell$$

where

$$\Lambda_{r_{1}}=\left\{ \begin{array}{cc} I & \text{ if }r_{1}=0\\ \prod_{i=0}^{r_{1}}P_{0,1}A_{i} & \text{ if }r_{1}=1,2,\cdots ,\ell \end{array}\right.$$

Substituting equations (18) and (25) in (22), we get

(26) $$\Pi^{\left(0\right)}[I+\sum_{r_{1}=1}^{\ell -1}\Lambda_{r_{1}}+\Lambda_{\ell }{\left(I-R\right)}^{-1}]\text{e}=1.$$

Solving (26) and (19), $\Pi^{\left(0\right)}$ can be calculated.

## 5 System performance metrics

Measuring system performance is essential for establishing objectives, coordinating efforts, allocating resources, and ultimately attaining organizational success. It provides a structured framework for administering and enhancing the performance of an organization's systems and processes. In this section, various performance measures of the system are established to construct the total cost function and to study the characteristics of the system.

1. **Expected number of customers in the waiting hall:**
  $E_{WH}=\sum_{r_{1}=0}^{\infty }\sum_{r_{3}=0}^{s}\sum_{r_{4}=1}^{S_{2}}\sum_{r_{6}=1}^{N}r_{6}\Pi^{\left(r_{1},0,r_{3},r_{4},0,r_{6}\right)}+\sum_{r_{1}=0}^{\infty }\sum_{r_{2}=1}^{s}\sum_{r_{3}=0}^{s-r_{2}}\sum_{r_{4}=1}^{S_{2}}\sum_{r_{5}=0,1}\sum_{r_{6}=1}^{N}r_{6}\Pi^{\left(r_{1},r_{2},r_{3},r_{4},r_{5},r_{6}\right)}$
  $+\sum_{r_{1}=0}^{\infty }\sum_{r_{2}=s+1}^{S_{1}}\sum_{r_{3}=0}^{S_{1}-r_{2}}\sum_{r_{4}=1}^{S_{2}}\sum_{r_{5}=0,1}\sum_{r_{6}=1}^{N}r_{6}\Pi^{\left(r_{1},r_{2},r_{3},r_{4},r_{5},r_{6}\right)}$
2. **Expected number of primary items:**
  $E_{I_{1}}=\sum_{r_{1}=0}^{\infty }\sum_{r_{2}=1}^{s}\sum_{r_{3}=0}^{s-r_{2}}\sum_{r_{4}=1}^{S_{2}}\sum_{r_{6}=0}^{N}r_{2}\Pi^{\left(r_{1},r_{2},r_{3},r_{4},0,r_{6}\right)}+\sum_{r_{1}=0}^{\infty }\sum_{r_{2}=1}^{s}\sum_{r_{3}=0}^{s-r_{2}}\sum_{r_{4}=1}^{S_{2}}\sum_{r_{6}=1}^{N}r_{2}\Pi^{\left(r_{1},r_{2},r_{3},r_{4},1,r_{6}\right)}$
  $+\sum_{r_{1}=0}^{\infty }\sum_{r_{2}=s+1}^{S_{1}}\sum_{r_{3}=0}^{S_{1}-r_{2}}\sum_{r_{4}=1}^{S_{2}}\sum_{r_{6}=0}^{N}r_{2}\Pi^{\left(r_{1},r_{2},r_{3},r_{4},0,r_{6}\right)}+\sum_{r_{1}=0}^{\infty }\sum_{r_{2}=s+1}^{S_{1}}\sum_{r_{3}=0}^{S_{1}-r_{2}}\sum_{r_{4}=1}^{S_{2}}\sum_{r_{6}=1}^{N}r_{2}\Pi^{\left(r_{1},r_{2},r_{3},r_{4},1,r_{6}\right)}$
3. **Expected number of complimentary items:**
  $E_{I_{2}}=\sum_{r_{1}=0}^{\infty }\sum_{r_{2}=0}^{s}\sum_{r_{3}=0}^{s-r_{2}}\sum_{r_{4}=1}^{S_{2}}\sum_{r_{6}=0}^{N}r_{4}\Pi^{\left(r_{1},r_{2},r_{3},r_{4},0,r_{6}\right)}+\sum_{r_{1}=0}^{\infty }\sum_{r_{2}=1}^{s}\sum_{r_{3}=0}^{s-r_{2}}\sum_{r_{4}=1}^{S_{2}}\sum_{r_{6}=1}^{N}r_{4}\Pi^{\left(r_{1},r_{2},r_{3},r_{4},1,r_{6}\right)}+$
  $\sum_{r_{1}=0}^{\infty }\sum_{r_{2}=s+1}^{S_{1}}\sum_{r_{3}=0}^{S_{1}-r_{2}}\sum_{r_{4}=1}^{S_{2}}\sum_{r_{6}=0}^{N}r_{4}\Pi^{\left(r_{1},r_{2},r_{3},r_{4},0,r_{6}\right)}+\sum_{r_{1}=0}^{\infty }\sum_{r_{2}=s+1}^{S_{1}}\sum_{r_{3}=0}^{S_{1}-r_{2}}\sum_{r_{4}=1}^{S_{2}}\sum_{r_{6}=1}^{N}r_{4}\Pi^{\left(r_{1},r_{2},r_{3},r_{4},1,r_{6}\right)}$
4. **Expected number of replaceable items in the pooled place:**
  $E_{RI}=\sum_{r_{1}=0}^{\infty }\sum_{r_{2}=0}^{s-1}\sum_{r_{3}=1}^{s-r_{2}}\sum_{r_{4}=1}^{S_{2}}\sum_{r_{6}=0}^{N}r_{3}\Pi^{\left(r_{1},r_{2},r_{3},r_{4},0,r_{6}\right)}+\sum_{r_{1}=0}^{\infty }\sum_{r_{2}=1}^{s-1}\sum_{r_{3}=1}^{s-r_{2}}\sum_{r_{4}=1}^{S_{2}}\sum_{r_{6}=1}^{N}r_{3}\Pi^{\left(r_{1},r_{2},r_{3},r_{4},1,r_{6}\right)}+$
  $\sum_{r_{1}=0}^{\infty }\sum_{r_{2}=s+1}^{S_{1}-1}\sum_{r_{3}=1}^{S_{1}-r_{2}}\sum_{r_{4}=1}^{S_{2}}\sum_{r_{6}=0}^{N}r_{3}\Pi^{\left(r_{1},r_{2},r_{3},r_{4},0,r_{6}\right)}+\sum_{r_{1}=0}^{\infty }\sum_{r_{2}=s+1}^{S_{1}-1}\sum_{r_{3}=1}^{S_{1}-r_{2}}\sum_{r_{4}=1}^{S_{2}}\sum_{r_{6}=1}^{N}r_{3}\Pi^{\left(r_{1},r_{2},r_{3},r_{4},1,r_{6}\right)}$
5. **Expected reorder rate for the primary item:**
  $E_{RR_{1}}=\sum_{r_{1}=0}^{\infty }\sum_{r_{3}=0}^{S_{1}-(s+1)}\sum_{r_{4}=1}^{S_{2}}\sum_{r_{6}=1}^{N}p_{1}\mu_{1}^{\prime }\Pi^{\left(r_{1},s+1,r_{3},r_{4},0,r_{6}\right)}+\sum_{r_{1}=0}^{\infty }\sum_{r_{3}=0}^{S_{1}-(s+1)}\sum_{r_{4}=1}^{S_{2}}\sum_{r_{6}=1}^{N}p_{1}\mu_{1}\Pi^{\left(r_{1},s+1,r_{3},r_{4},1,r_{6}\right)}$
  $+\sum_{r_{1}=0}^{\infty }\sum_{r_{3}=0}^{S_{1}-(s+1)}\sum_{r_{4}=1}^{S_{2}}(s+1)\gamma_{1}\Pi^{\left(r_{1},s+1,r_{3},r_{4},0,0\right)}+\sum_{r_{1}=0}^{\infty }\sum_{r_{3}=0}^{S_{1}-(s+1)}\sum_{r_{4}=1}^{S_{2}}\sum_{r_{5}=0,1}\sum_{r_{6}=1}^{N}(s+1)\gamma_{1}\Pi^{\left(r_{1},s+1,r_{3},r_{4},r_{5},r_{6}\right)}$
6. **Expected reorder rate for the complimentary item:**
  $E_{RR_{2}}=\sum_{r_{1}=0}^{\infty }\sum_{r_{3}=0}^{s}\sum_{r_{6}=0}^{N}\gamma_{2}\Pi^{\left(r_{1},0,r_{3},1,0,r_{6}\right)}+\sum_{r_{1}=0}^{\infty }\sum_{r_{2}=1}^{s}\sum_{r_{3}=0}^{s-r_{2}}\sum_{r_{6}=0}^{N}\gamma_{2}\Pi^{\left(r_{1},r_{2},r_{3},1,0,r_{6}\right)}+$
  $\sum_{r_{1}=0}^{\infty }\sum_{r_{2}=1}^{s}\sum_{r_{3}=0}^{s-r_{2}}\sum_{r_{6}=1}^{N}\gamma_{2}\Pi^{\left(r_{1},r_{2},r_{3},1,1,r_{6}\right)}+\sum_{r_{1}=0}^{\infty }\sum_{r_{2}=s+1}^{S_{1}}\sum_{r_{3}=0}^{S_{1}-r_{2}}\sum_{r_{6}=0}^{N}\gamma_{2}\Pi^{\left(r_{1},r_{2},r_{3},1,0,r_{6}\right)}+$
  $\sum_{r_{1}=0}^{\infty }\sum_{r_{2}=s+1}^{S_{1}}\sum_{r_{3}=0}^{S_{1}-r_{2}}\sum_{r_{6}=1}^{N}\gamma_{2}\Pi^{\left(r_{1},r_{2},r_{3},1,1,r_{6}\right)}+\sum_{r_{1}=0}^{\infty }\sum_{r_{3}=0}^{s}\sum_{r_{6}=1}^{N}p_{2}\mu_{2}^{\prime }\Pi^{\left(r_{1},r_{2},r_{3},1,0,r_{6}\right)}+$
  $\sum_{r_{1}=0}^{\infty }\sum_{r_{2}=1}^{s}\sum_{r_{3}=0}^{s-r_{2}}\sum_{r_{6}=1}^{N}(p_{1}\mu_{1}^{\prime }+p_{2}\mu_{2}^{\prime })\Pi^{\left(r_{1},r_{2},r_{3},1,0,r_{6}\right)}+\sum_{r_{1}=0}^{\infty }\sum_{r_{2}=1}^{s}\sum_{r_{3}=0}^{s-r_{2}}\sum_{r_{6}=1}^{N}(p_{1}\mu_{1}+p_{2}\mu_{2})\Pi^{\left(r_{1},r_{2},r_{3},1,1,r_{6}\right)}+$
  $\sum_{r_{1}=0}^{\infty }\sum_{r_{2}=s+1}^{S_{1}}\sum_{r_{3}=0}^{S_{1}-r_{2}}\sum_{r_{6}=1}^{N}(p_{1}\mu_{1}^{\prime }+p_{2}\mu_{2}^{\prime })\Pi^{\left(r_{1},r_{2},r_{3},1,0,r_{6}\right)}+\sum_{r_{1}=0}^{\infty }\sum_{r_{2}=s+1}^{S_{1}}\sum_{r_{3}=0}^{S_{1}-r_{2}}\sum_{r_{6}=1}^{N}(p_{1}\mu_{1}+p_{2}\mu_{2})\Pi^{\left(r_{1},r_{2},r_{3},1,1,r_{6}\right)}$
7. **Expected perishable rate for the primary item:**
  $E_{PR_{1}}=\gamma_{1}E_{I_{1}}$
8. **Expected number of customers in the orbit:**
  $E_{O}=(\sum_{r_{1}=1}^{\ell }r_{1}\Pi^{\left(r_{1}\right)}+\Pi^{\left(\ell \right)}[\ell {\left(I-R\right)}^{-1}+R{\left(I-R\right)}^{-2}]).\text{e}$
9. **Expected rate at which a customer lost happens:**
  $E_{L}=\sum_{r_{1}=0}^{\infty }\sum_{r_{3}=0}^{s}\sum_{r_{4}=1}^{S_{2}}(1-r)\lambda_{0}\Pi^{\left(r_{1},0,r_{3},r_{4},0,N\right)}+\sum_{r_{1}=0}^{\infty }\sum_{r_{2}=1}^{s}\sum_{r_{3}=0}^{s-r_{2}}\sum_{r_{4}=1}^{S_{2}}\sum_{r_{5}=0,1}(1-r)\lambda_{j}\Pi^{\left(r_{1},r_{2},r_{3},r_{4},r_{5},N\right)}+$
  $\sum_{r_{1}=0}^{\infty }\sum_{r_{2}=s+1}^{S_{1}}\sum_{r_{3}=0}^{S_{1}-r_{2}}\sum_{r_{4}=1}^{S_{2}}\sum_{r_{5}=0,1}(1-r)\lambda_{j}\Pi^{\left(r_{1},r_{2},r_{3},r_{4},r_{5},N\right)}$
10. **Expected rate at which a customer in the waiting hall do not buy any item:**
  $$E_{NB}=\sum_{r_{1}=0}^{\infty }\sum_{r_{2}=0}^{s}\sum_{r_{3}=0}^{s-r_{2}}\sum_{r_{4}=1}^{S_{2}}\sum_{r_{6}=1}^{N}(p_{3}+p_{4})\mu_{3}^{\prime }\Pi^{\left(r_{1},r_{2},r_{3},r_{4},0,r_{6}\right)}+\;\;\;\;\;\;\;\;\;\;\;\;\;\;\sum_{r_{1}=0}^{\infty }\sum_{r_{2}=0}^{s}\sum_{r_{3}=0}^{s-r_{2}}\sum_{r_{4}=1}^{S_{2}}\sum_{r_{6}=1}^{N}(p_{3}+p_{4})\mu_{3}\Pi^{\left(r_{1},r_{2},r_{3},r_{4},1,r_{6}\right)}+\;\;\;\;\;\;\;\;\;\;\;\;\;\;\sum_{r_{1}=0}^{\infty }\sum_{r_{2}=s+1}^{S_{1}}\sum_{r_{3}=0}^{S_{1}-r_{2}}\sum_{r_{4}=1}^{S_{2}}\sum_{r_{6}=1}^{N}(p_{3}+p_{4})\mu_{3}^{\prime }\Pi^{\left(r_{1},r_{2},r_{3},r_{4},0,r_{6}\right)}+\;\;\;\;\;\;\;\;\;\;\;\;\;\;\sum_{r_{1}=0}^{\infty }\sum_{r_{2}=s+1}^{S_{1}}\sum_{r_{3}=0}^{S_{1}-r_{2}}\sum_{r_{4}=1}^{S_{2}}\sum_{r_{6}=1}^{N}(p_{3}+p_{4})\mu_{3}\Pi^{\left(r_{1},r_{2},r_{3},r_{4},1,r_{6}\right)}$$
11. **Fraction of time server is on working vacation mode:**
  $FT_{wv}=\sum_{r_{1}=0}^{\infty }\sum_{r_{2}=0}^{s}\sum_{r_{3}=0}^{s-r_{2}}\sum_{r_{4}=1}^{S_{2}}\sum_{r_{6}=0}^{N}\Pi^{\left(r_{1},r_{2},r_{3},r_{4},0,r_{6}\right)}+\sum_{r_{1}=0}^{\infty }\sum_{r_{2}=1}^{s}\sum_{r_{3}=0}^{s-r_{2}}\sum_{r_{4}=1}^{S_{2}}\sum_{r_{6}=1}^{N}\Pi^{\left(r_{1},r_{2},r_{3},r_{4},1,r_{6}\right)}$
  $\sum_{r_{1}=0}^{\infty }\sum_{r_{2}=s+1}^{S_{1}}\sum_{r_{3}=0}^{S_{1}-r_{2}}\sum_{r_{4}=1}^{S_{2}}\sum_{r_{6}=0}^{N}\Pi^{\left(r_{1},r_{2},r_{3},r_{4},0,r_{6}\right)}+\sum_{r_{1}=0}^{\infty }\sum_{r_{2}=s+1}^{S_{1}}\sum_{r_{3}=0}^{S_{1}-r_{2}}\sum_{r_{4}=1}^{S_{2}}\sum_{r_{6}=1}^{N}\Pi^{\left(r_{1},r_{2},r_{3},r_{4},1,r_{6}\right)}$

### 5.1 Expected total cost

The expected total expenditure of the considered QIS is defined as

$$\begin{array}{c} E_{TC}=c_{h_{1}}E_{I_{1}}+c_{h_{2}}E_{I_{2}}+c_{cp}E_{RI}+c_{s_{1}}E_{RR_{1}}+c_{s_{2}}E_{RR_{2}}+c_{p_{1}}E_{PR_{1}}+\\ c_{p_{2}}E_{RR_{2}}+c_{wh}E_{WH}+c_{s}E_{NB}+c_{o}E_{O}+c_{l}E_{L} \end{array}$$

where

$c_{s_{1}}$ - set-up cost per order for commodity-1 per unit time

$c_{s_{2}}$ - set-up cost per order for commodity-2 per unit time

$c_{h_{1}}$ - holding cost per unit item for commodity-1 per unit time

$c_{h_{2}}$ - holding cost per unit item for commodity-2 per unit time

$c_{cp}$ - carrying cost per unit perishable item in the pooled storage per unit time

$c_{p_{1}}$ - perishable cost per unit item for commodity-1 per unit time

$c_{p_{2}}$ - perishable cost per unit item for commodity-2 per unit time

$c_{wh}$ - waiting cost per customer in the waiting hall per unit time

$c_{s}$ - service cost per customer per unit time

$c_{o}$ - waiting cost per customer in the orbit per unit time

$c_{l}$ - loss cost per customer per unit time

## 6 Numerical analysis

Numerical analysis is indispensable to mathematical modeling because it provides the tools and techniques necessary to solve complex, real-world problems frequently intractable by purely analytical means. It enables researchers and engineers to obtain insights, make predictions, and optimize systems across various disciplines. In this section, the performance and characteristics of the model are analyzed numerically. The maximum capacity of the storage space for the first and second commodities are $S_{1}=10$ and $S_{2}=4$, respectively. We randomly divide the inventory into three levels such that $k_{1}=4,k_{2}=6$, and $k_{3}=10$. We assume N=6,n=2 and s=3. The parameter, probability and the cost values are assumed as $p_{1}=0.25,p_{2}=0.25,p_{3}=0.1,p_{4}=0.4,\ell =30,\mu_{1}=7,\mu_{1}^{\prime }=3,\mu_{2}=13,\mu_{2}^{\prime }=9,\mu_{3}=16,\mu_{3}^{\prime }=14,a_{1}=0.01,b_{1}=0.02,\eta =5,\beta =5,\gamma_{1}=0.1,\gamma_{2}=1,p=0.7,\lambda_{0}=0.7,\theta_{0}=0.01,\theta =0.08,\lambda =0.8,q=0.5,c_{h_{1}}=0.03,c_{h_{2}}=0.02,c_{cp}=0.01,c_{s_{1}}=0.5,c_{s_{2}}=0.1,c_{p_{1}}=0.02,c_{p_{2}}=0.01,c_{wh}=0.6,c_{o}=0.36$ and $c_{l}=5$. One of the key aspects of numerical computation is to make sure the results have internal accuracy checks. It has been carried out and verified as the details given in [46], [48], and [49].

In this numerical investigation, we have undertaken two comparison analyses and the parametric analysis of some key system performances. 1. The comparison between the replacement of failed items and the non-replacement of failed items. 2. The comparison between multi-component demand vs homogeneous arrival. The parametric analysis focuses on the number of customers in the waiting hall and orbit, the number of failed items in the pooled storage, and the fraction of time the server is on a working vacation.

### 6.1 Comparative analysis

#### 6.1.1 Comparison of expected total cost: replacement vs. non-replacement

In this section, we study the comparison of the model with replacement and without replacement of failed items compared with using the expected total cost through Figure 3, Figure 4, Figure 5, Figure 6, Figure 7, Figure 8, Figure 9, Figure 10, Figure 11, Figure 12, Figure 13, Figure 14.

**Figure 3:**
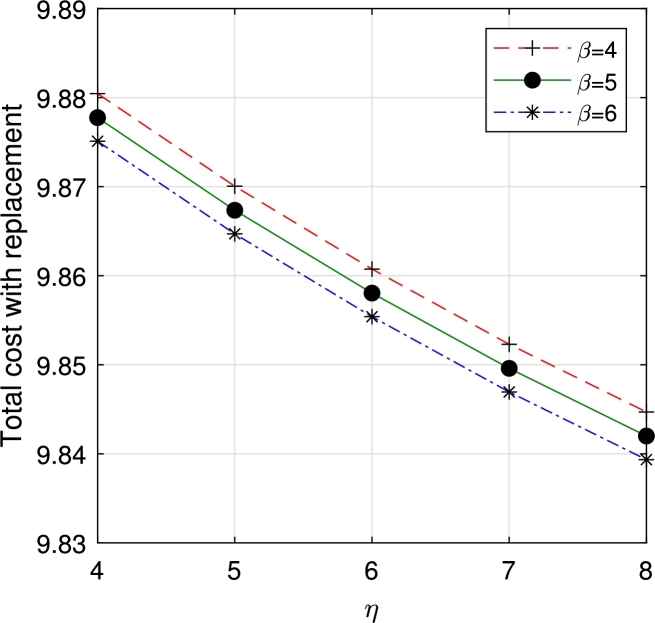
Total cost with replacement *β* vs *η*.

**Figure 4:**
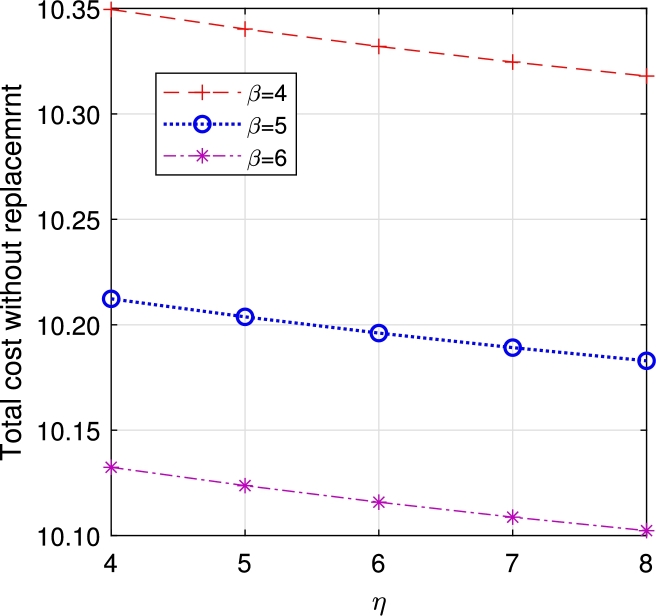
Total cost without replacement *β* vs *η*.

**Figure 5:**
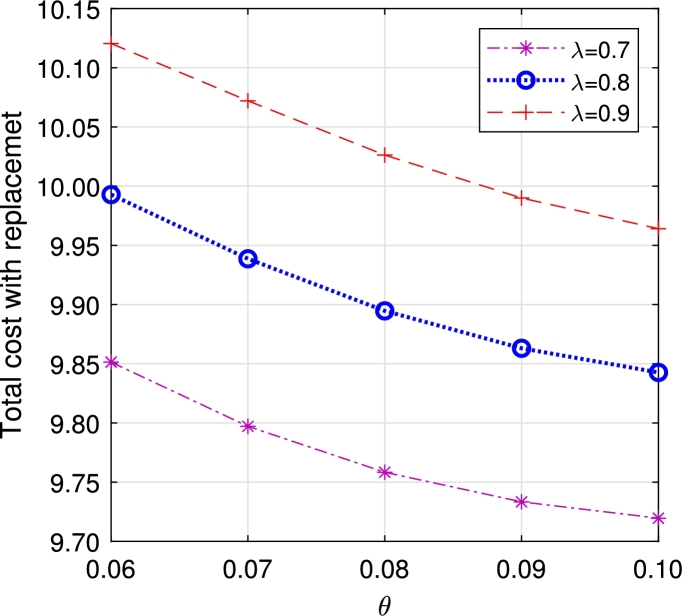
Total cost with replacement *λ* vs *θ*.

**Figure 6:**
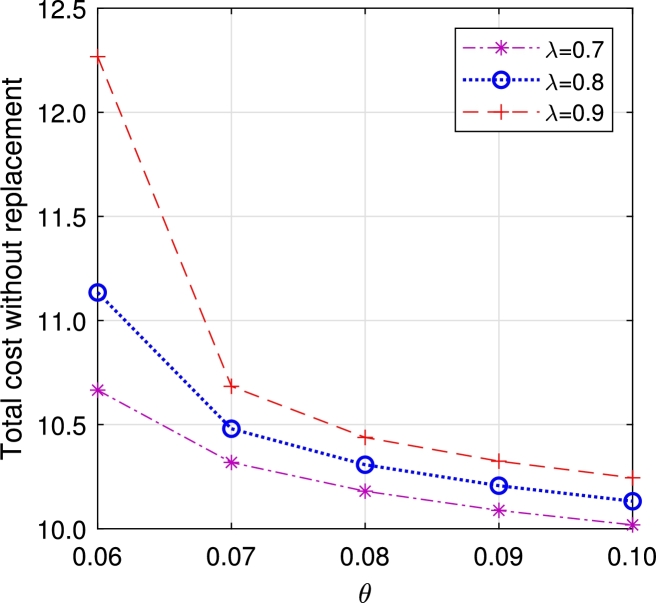
Total cost without replacement *λ* vs *θ*.

**Figure 7:**
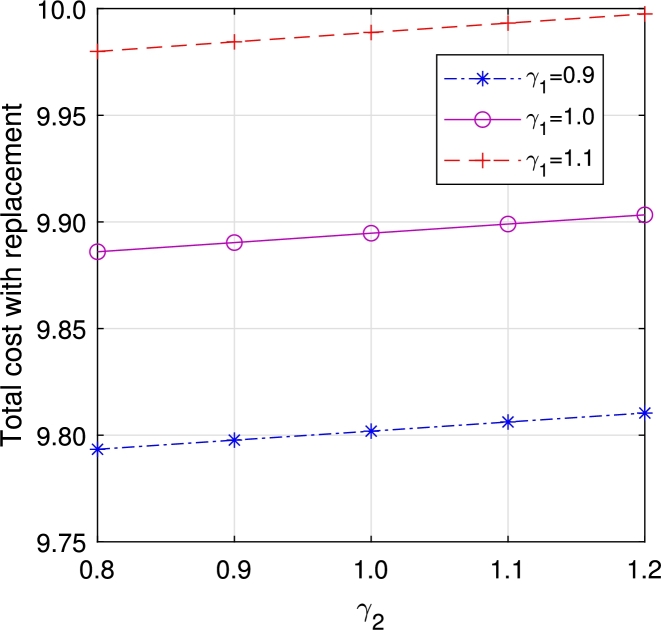
Total cost with replacement *γ*_1_ vs *γ*_2_.

**Figure 8:**
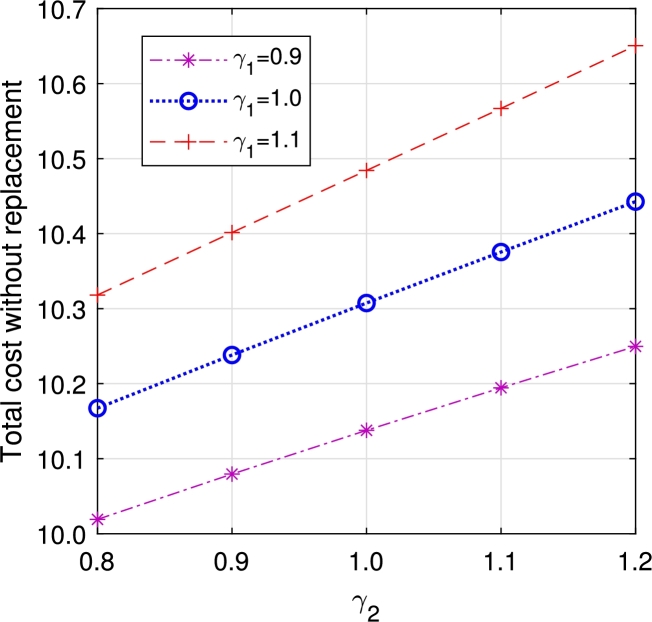
Total cost without replacement *γ*_1_ vs *γ*_2_.

**Figure 9:**
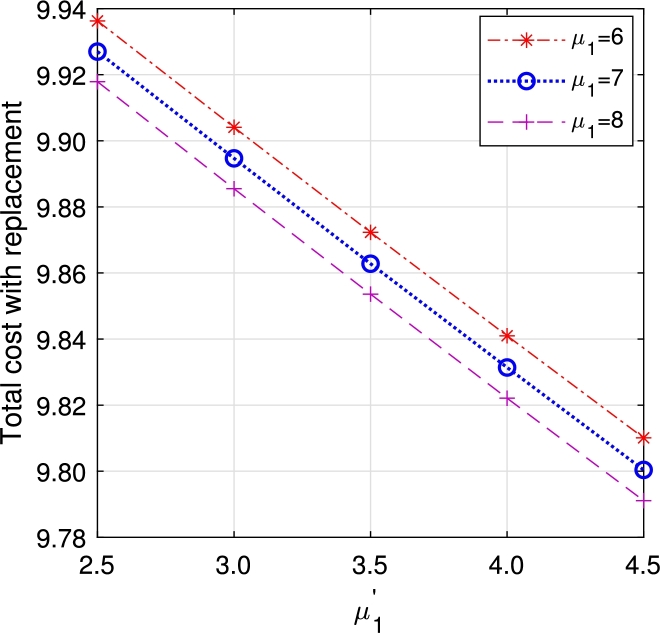
Total cost with replacement *μ*_1_ vs $\mu_{1}^{\prime }$.

**Figure 10:**
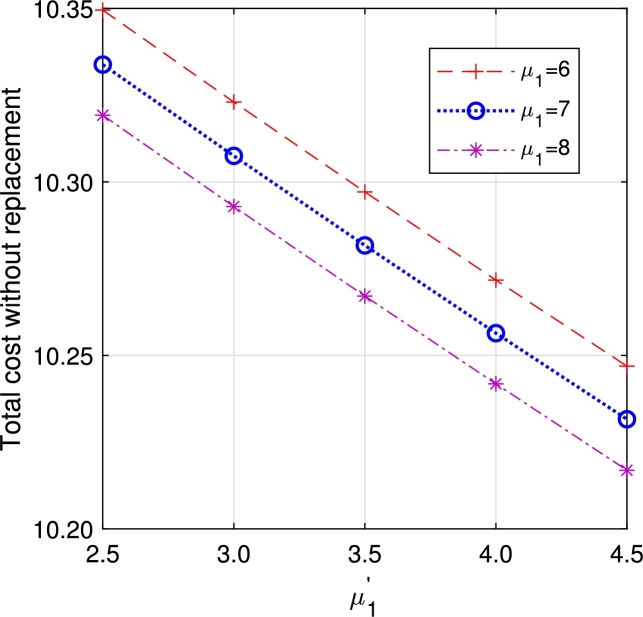
Total cost without replacement *μ*_1_ vs $\mu_{1}^{\prime }$.

**Figure 11:**
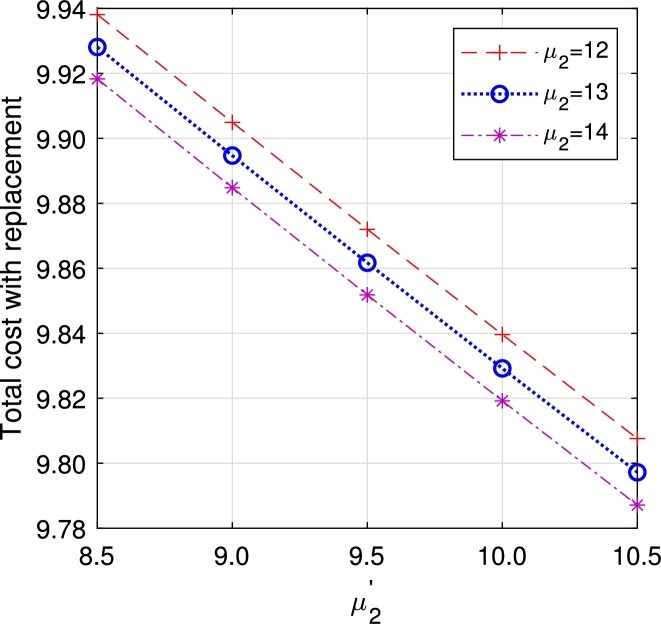
Total cost with replacement *μ*_2_ vs $\mu_{2}^{\prime }$.

**Figure 12:**
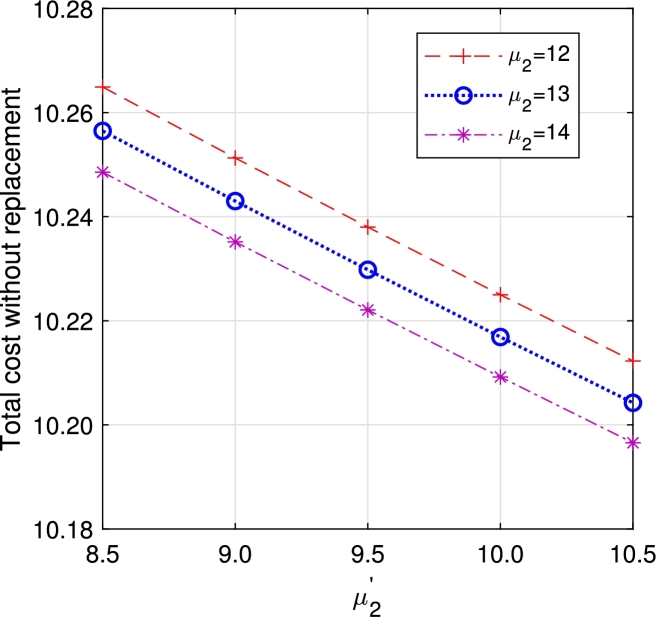
Total cost without replacement *μ*_2_ vs $\mu_{2}^{\prime }$.

**Figure 13:**
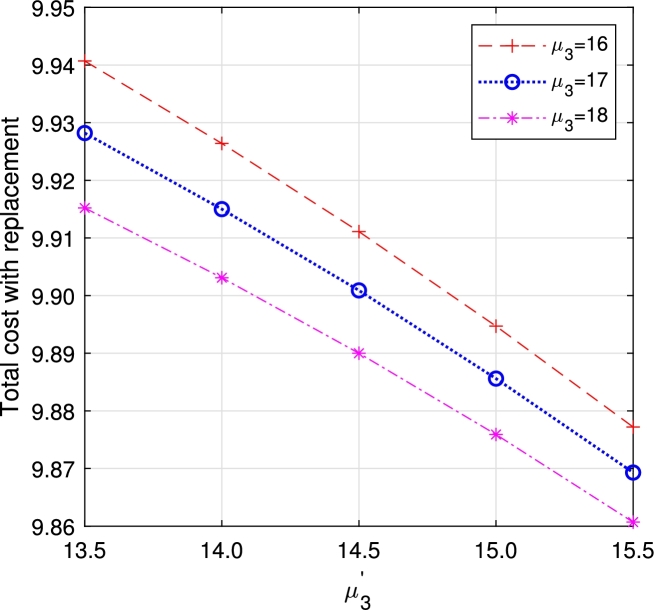
Total cost with replacement *μ*_3_ vs $\mu_{3}^{\prime }$.

**Figure 14:**
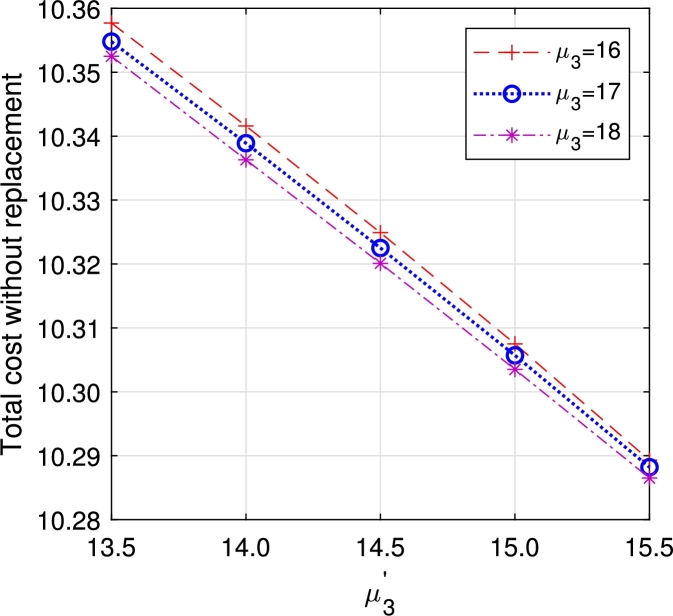
Total cost without replacement *μ*_3_ vs $\mu_{3}^{\prime }$.

We obtain the QIS without the replacement facility by modifying the level-dependent QBD process {R(t):t≥0} as removing the random variable $R_{3}$, which results the five-dimensional LDQBD with the state space $E^{\prime }=E_{1}^{\prime }\cup E_{2}^{\prime }$, where

$$E_{1}^{\prime }=\{(r_{1},r_{2},r_{4},r_{5},r_{6})|r_{1}\in N,r_{2}\in G_{0}^{S_{1}},r_{4}\in G_{1}^{S_{2}},r_{5}=0,r_{6}\in G_{0}^{N}\}$$

$$E_{2}^{\prime }=\{(r_{1},r_{2},r_{4},r_{5},r_{6})|r_{1}\in N,r_{2}\in G_{1}^{s},r_{4}\in G_{1}^{S_{2}},r_{5}=1,r_{6}\in G_{1}^{N}\}$$

This system also has the same structure as in Equation (1). So, the steady-state probability vector can be computed using Neuts and Rao's truncation method [46] as derived earlier.

• Figure 3, Figure 4 demonstrate how adjustments in the parameters *η* and *β* influence $E_{TC}$. Reducing both vacation and lead time leads to a decrease in $E_{TC}$. However, the cost fluctuations in the figures highlight that the model with replacement yields the lowest total cost compared to the option without replacement.
• In Figure 5, Figure 6, we observe the influence of primary and retrial arrivals on $E_{TC}$. In both scenarios, an increase in arrival rate leads to higher $E_{TC}$, while a decrease in $E_{O}$ coupled with an increase in *θ* results in a decrease. However, the findings highlight the cost-effectiveness of a replacement facility to manage $E_{TC}$.
• Figure 7, Figure 8 illustrate how perishable rates impact overall expenses. When an item expires, it leads to financial losses for the business. Nonetheless, a comparison demonstrates that replacing the faulty item proves instrumental in mitigating such losses.
• Figure 9, Figure 10, Figure 11, Figure 12, Figure 13, Figure 14 scrutinize the influence of service rates ($\mu_{k}$ and $\mu_{k}^{\prime }$ for k=1,2,3) on $E_{TC}$. Elevating the service rate reduces service duration, aiding in the reduction of $E_{WH}$ and subsequently lowering $E_{TC}$. Across all comparisons, a system equipped with a replacement facility consistently outperforms one without it in terms of efficiency.

The comparative study reveals the efficiency of the replacement of failed items. Replacing failed items is a critical practice in various real-life engineering settings. Here are some common applications: In the aerospace industry, it is customary to regularly inspect and replace vital elements like engines and avionics systems in order to prevent any catastrophic malfunctions. Similarly, in automobile engineering, the routine replacement of old or broken components is considered a customary maintenance procedure. This notion applies to industrial operations, whereby machinery and production line equipment are regularly updated to maintain consistent and efficient production processes.

In the grocery and food industry, the expeditious replacement of expired or ruined products is essential to maintain consumer happiness and adhere to food safety regulations. In the retail industry, it is important to prioritize the timely replacement of defective items such as cell phones and home appliances to maintain high levels of customer happiness and safeguard the brand's image for excellence. Furthermore, it is essential for enterprises operating in the pharmaceutical and personal care industries to expeditiously replace items that have reached their expiration date or have incurred damage in order to safeguard the safety and welfare of their customers.

Strategic and timely replacement of failed items helps businesses maintain operational efficiency, uphold quality standards, reduce costs associated with downtime and repairs, and ultimately enhance customer satisfaction and loyalty.

#### 6.1.2 Comparison of multi-component demand rate with homogeneous arrival rate

In this section, comparison of multi-component demand and homogeneous demand rates are compared over $E_{TC}$. The following characteristics are observed from the study.

**Figure 15:**
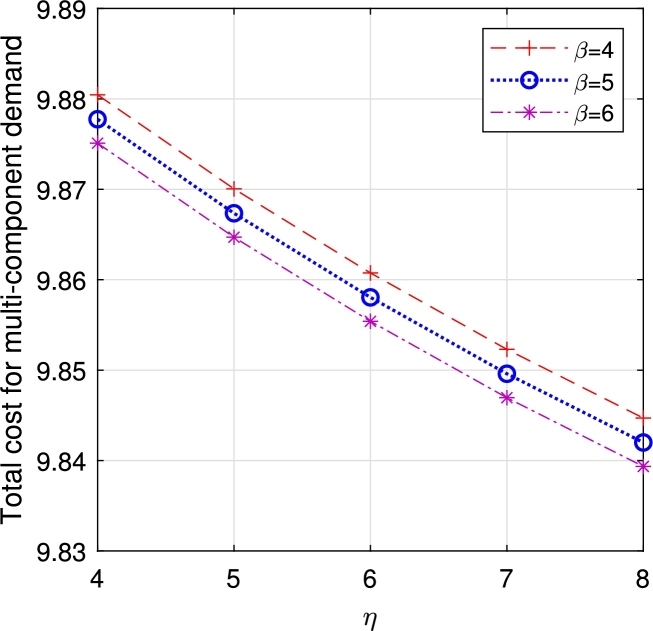
Total cost for multi-component demand *β* vs *η*.

**Figure 16:**
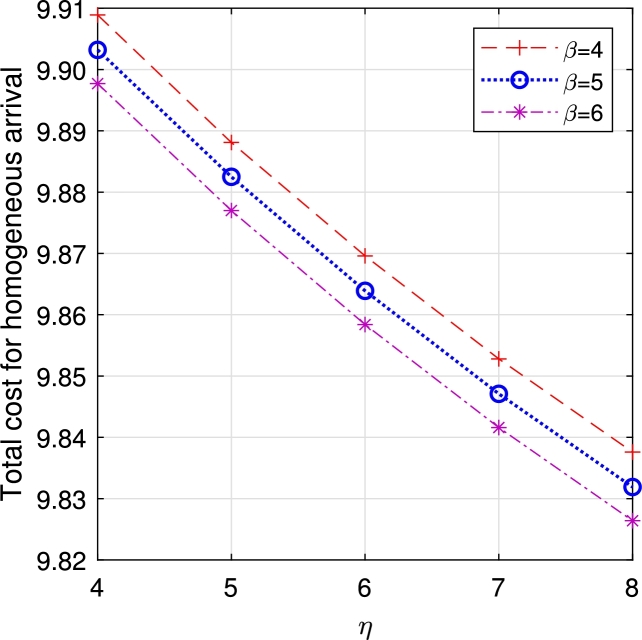
Total cost for homogeneous arrival *β* vs *η*.
• The Figure 17, Figure 18 depict the influence of retrial and arrival rates. When the rate *λ* rises, there is an increase in $E_{TC}$ due to a rise in $E_{WH}$. Similarly, an increase in the rate *θ* leads to a reduction in orbital customers, subsequently decreasing $E_{TC}$.

• Figure 15, Figure 16 analyze the characteristics of *η* and *β*. As these parameters increase, both vacation duration and order lead time decrease, resulting in a reduction of $E_{TC}$.

**Figure 17:**
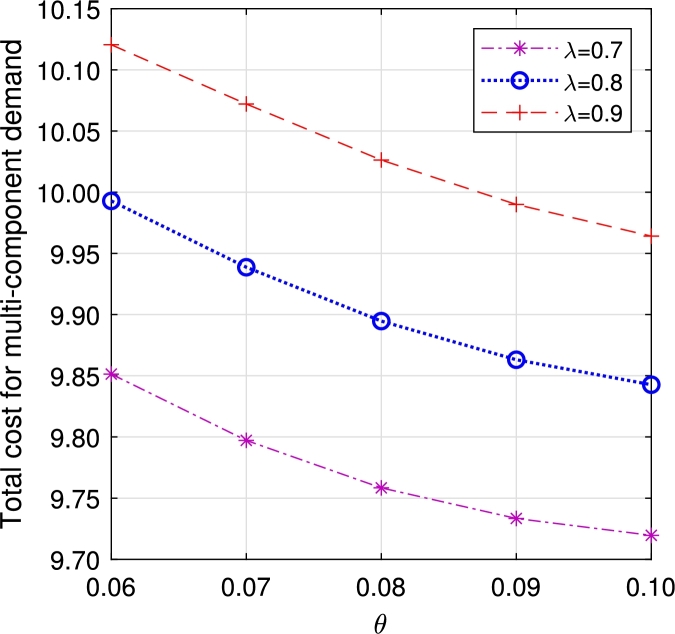
Total cost for multi-component demand *λ* vs *θ*.

**Figure 18:**
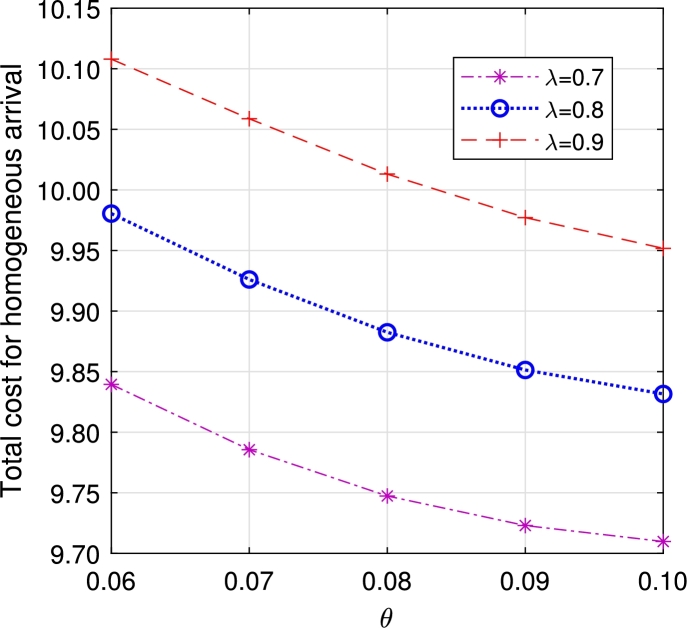
Total cost for homogeneous arrival *λ* vs *θ*.
• The impact of perishability on $E_{TC}$ is illustrated in Figure 19, Figure 20, considering both multi-component demand and homogeneous arrival, respectively. In the event of an item failure, it results in a loss to the system, leading to an increase in $E_{TC}$ in both cases.

**Figure 19:**
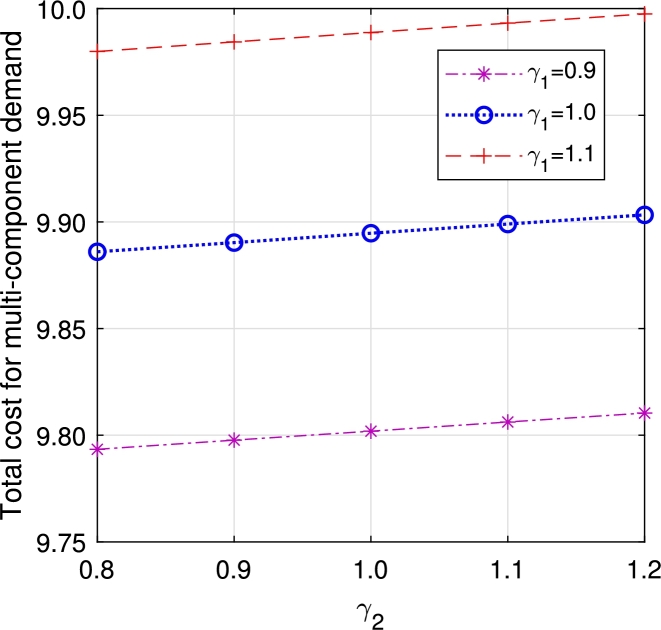
Total cost for multi-component demand *γ*_1_ vs *γ*_2_.

**Figure 20:**
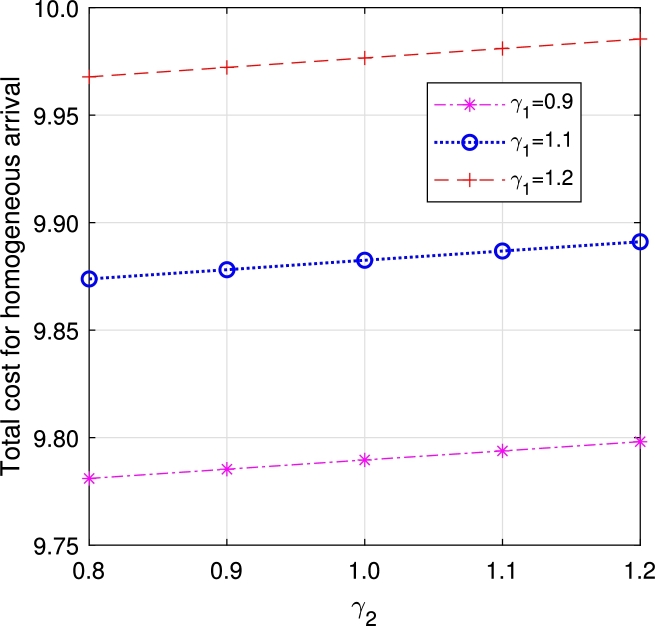
Total cost for homogeneous arrival *γ*_1_ vs *γ*_2_.
• Figure 21, Figure 22, Figure 23, Figure 24, Figure 25, Figure 26 demonstrate a decrease in $E_{TC}$ with an increase in service rates, both in regular server mode and working vacation mode. This reduction is attributed to the faster service of the server, which in turn reduces $E_{WH}$.

**Figure 21:**
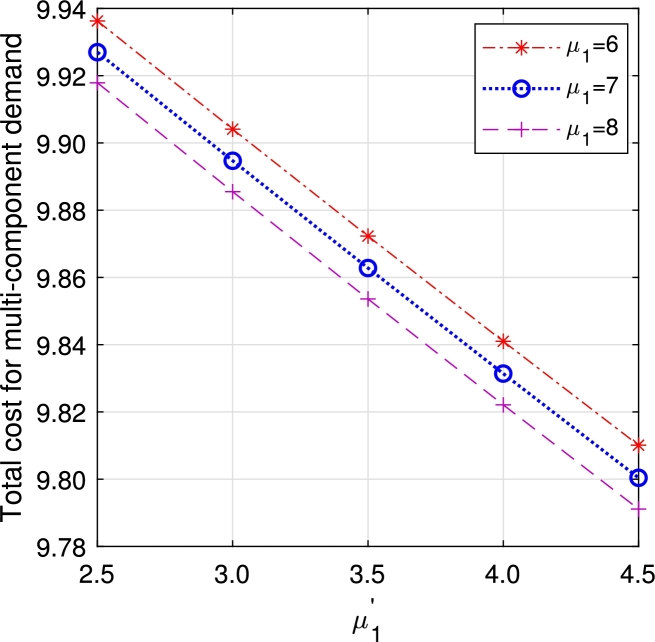
Total cost for multi-component demand arrival *μ*_1_ vs $\mu_{1}^{\prime }$.

**Figure 22:**
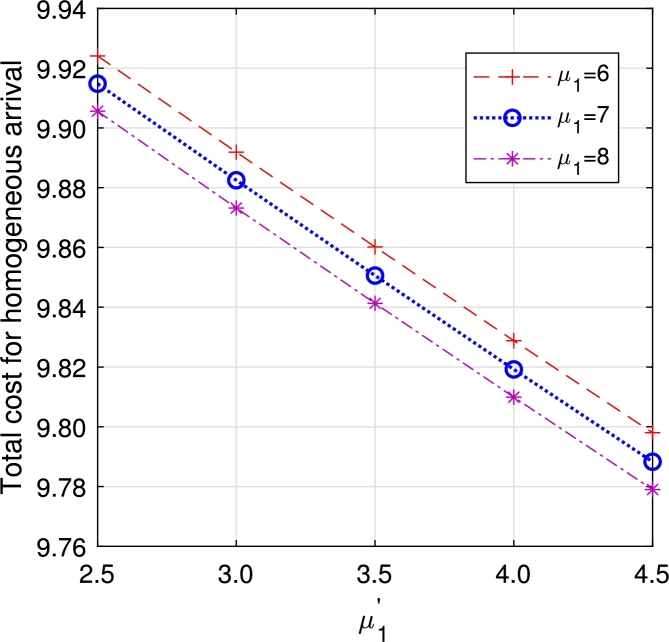
Total cost for homogeneous arrival *μ*_1_ vs $\mu_{1}^{\prime }$.

**Figure 23:**
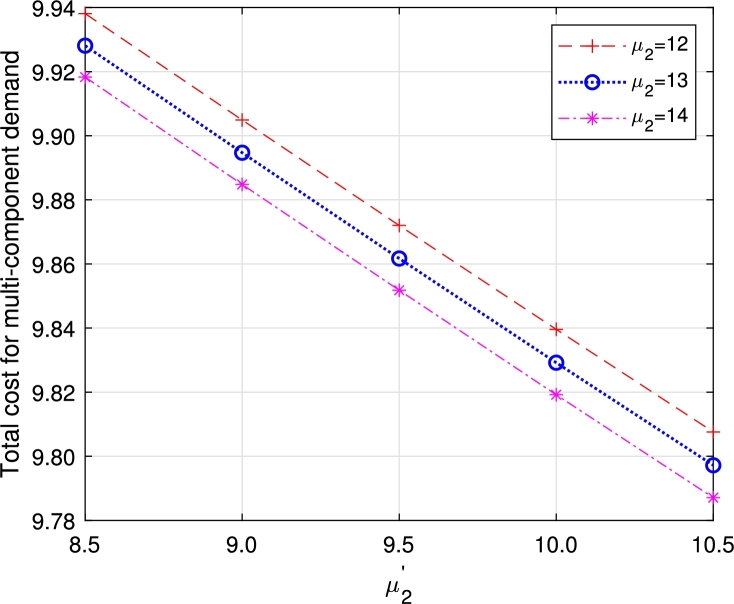
Total cost for multi-component demand *μ*_2_ vs $\mu_{2}^{\prime }$.

**Figure 24:**
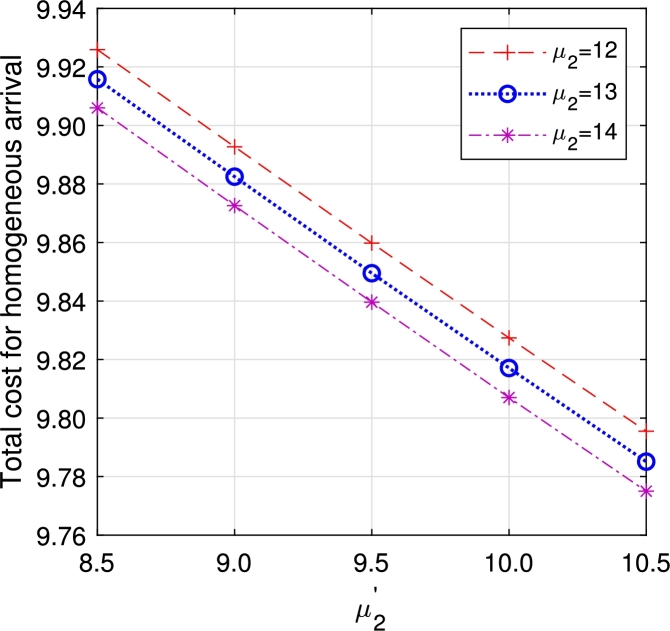
Total cost for homogeneous arrival *μ*_2_ vs $\mu_{2}^{\prime }$.

**Figure 25:**
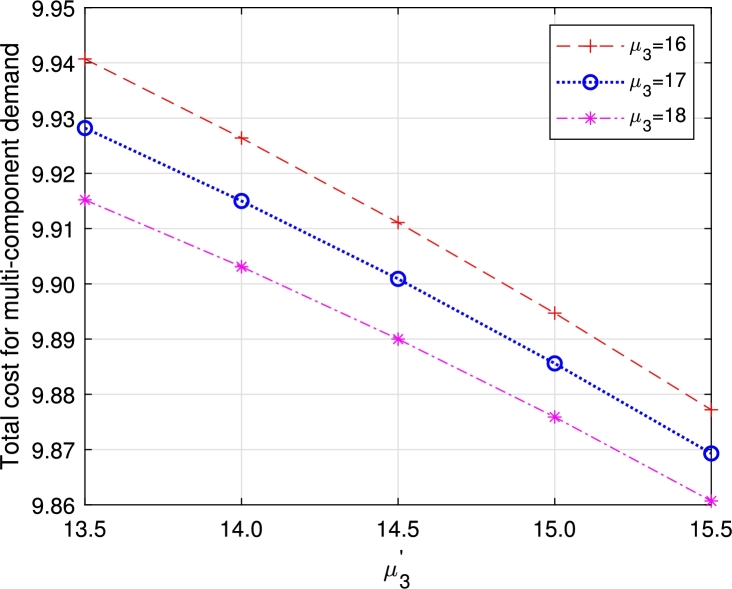
Total cost for multi-component demand *μ*_3_ vs $\mu_{3}^{\prime }$.

**Figure 26:**
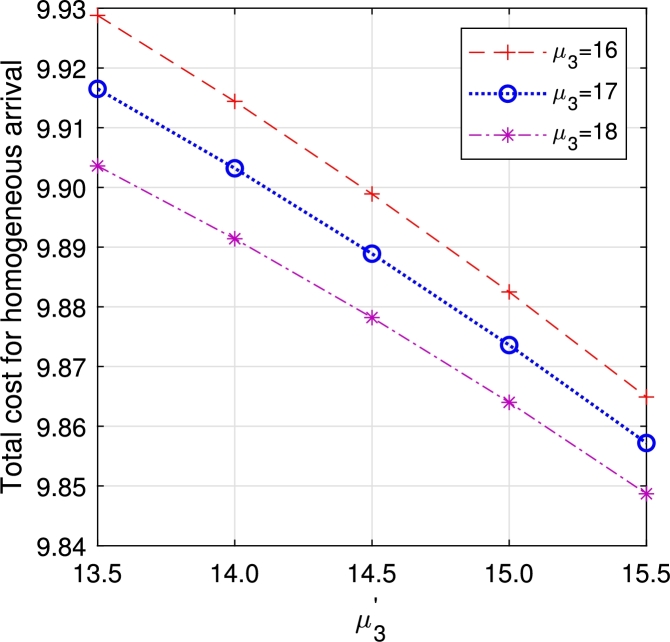
Total cost for homogeneous arrival *μ*_3_ vs $\mu_{3}^{\prime }$.

From the observation, though all the parameters react on the $E_{TC}$ with their unique characteristics, the one with multi-component demand attains total cost relatively higher than the one with homogeneous demand rate, which guarantees the arrival of customers due to the attraction on displayed items. In a business, customer arrival increases the total expenditure, but in return, it gains more profit for the system. In the retail sector, the number of items, especially during sales or promotions, can significantly impact customer traffic. Availability of popular products tends to increase customer arrivals, influencing queue lengths and service times. This phenomenon is also observed in online retail, where the availability of specific items can lead to surges in website traffic and order volumes. Similarly, in distribution, the availability of certain products can cause fluctuations in customer orders, with the presence of specific items leading to higher order volumes.

Implementing multi-component demand in practical settings is subject to variation, contingent upon variables such as data availability, industry-specific obstacles, the use of technology and software, and the amount of knowledge within the organization. The installation process for organizations with sophisticated inventory management systems and pertinent data might be simple.

### 6.2 Parametric sensitivity analysis: $E_{WH},\;E_{O},\;E_{RI}$ and $FT_{wv}$

#### 6.2.1 Analysis of mean number of customers in the waiting hall

In this section, the mean number of customers in the waiting hall is analyzed as it is directly proportional to the waiting time; it would be more helpful to analyze the waiting time. After observing the Table 2, Table 3, Table 4, the following characteristics are revealed.

• As the arrival rate increases, customers experience longer waiting times. That is because a higher arrival rate shortens the time between consecutive customer arrivals, increasing $E_{WH}$, which is shown in Table 2.
• Similarly, when the rate *θ* rises, the number of customers from the orbit decreases as they enter the waiting area. Consequently, $E_{WH}$ increases in Table 2.
• In Table 2, increasing the rate *η* leads to a quicker transition from server vacation to regular service mode that helps alleviate congestion in the waiting area.
• With an increase in replenishment rate *β*, lead times shorten. That means customers do not have to wait as long in the system due to stock-outs, ultimately reducing $E_{WH}$ in Table 2.
• The service rates during both vacation and regular periods exhibit similar effects on the system. When service rates increase, the average service time per customer decreases. As a result, customers spend less time in the system, leading to a decrease in $E_{WH}$ as service rates increase, which is shown in Table 3, Table 4.
• From Table 4, perishable rates have an inversely proportional effect on $E_{WH}$, as opposed to service rates. When perishable rates increase, items in the system perish more quickly, leading to more frequent stock-outs and longer customer wait times.

Studying the number of customers in the waiting area is a flexible method used in various industries to enhance operational effectiveness, elevate customer contentment, and fine-tune resource distribution. Furthermore, it is evident from those, as mentioned earlier, that certain factors impact managing congestion in the waiting hall.

**Table 2:** Impact of *β*, *λ*, *η* and *θ* on *E*_*WH*_.

*β*	*λ*	*η*	4			5			6		
		*θ*	0.06	0.08	0.10	0.06	0.08	0.10	0.06	0.08	0.10
4	0.6	0.29060		0.37150	0.45750	0.28740	0.36730	0.45230	0.28450	0.36350	0.44760
	0.8	0.32750		0.41260	0.50210	0.32370	0.40770	0.49620	0.32030	0.40340	0.49100
	1.0	0.36440		0.45500	0.54860	0.36010	0.44950	0.54200	0.35620	0.44460	0.53610

5	0.6	0.29049		0.37130	0.45719	0.28730	0.36710	0.45200	0.28438	0.36336	0.44736
	0.8	0.32734		0.41235	0.50178	0.32360	0.40750	0.49590	0.32020	0.40320	0.49065
	1.0	0.36420		0.45469	0.54814	0.35990	0.44920	0.54160	0.35607	0.44431	0.53567

6	0.6	0.29030		0.37100	0.45670	0.28710	0.36680	0.45150	0.28420	0.36310	0.44690
	0.8	0.32710		0.41200	0.50120	0.32340	0.40710	0.49530	0.32000	0.40280	0.49010
	1.0	0.36390		0.45420	0.54740	0.35960	0.44880	0.54080	0.35580	0.44390	0.53500

**Table 3:** Impact of $\mu_{1},\;\mu_{1}^{\prime },\;\mu_{2}$ and $\mu_{2}^{\prime }$ on *E*_*WH*_.

*μ*_1_	*μ*_2_	$\mu_{1}^{\prime }$	2			3			4		
		$\mu_{2}^{\prime }$	8	9	10	8	9	10	8	9	10
6	12	0.43780		0.42659	0.41594	0.42660	0.41600	0.40590	0.41606	0.40594	0.39633
	13	0.43327		0.42215	0.41158	0.42220	0.41160	0.40160	0.41170	0.40167	0.39214
	14	0.42899		0.41794	0.40746	0.41800	0.40750	0.39760	0.40758	0.39762	0.38817

7	12	0.43330		0.42210	0.41160	0.42220	0.41160	0.40160	0.41170	0.40170	0.39210
	13	0.42900		0.41790	0.40750	0.41800	0.40750	0.39760	0.40760	0.39760	0.38820
	14	0.42490		0.41400	0.40360	0.41400	0.40360	0.39370	0.40370	0.39380	0.38440

8	12	0.42900		0.41790	0.40750	0.41800	0.40750	0.39760	0.40758	0.39762	0.38816
	13	0.42490		0.41400	0.40360	0.41400	0.40360	0.39370	0.40368	0.39379	0.38440
	14	0.42110		0.41020	0.39980	0.41020	0.39990	0.39010	0.39998	0.39016	0.38083

**Table 4:** Impact of $\mu_{3},\;\mu_{3}^{\prime },\;\gamma_{1}$ and *γ*_2_ on *E*_*WH*_.

*γ*_1_	*μ*_3_	$\gamma_{2}$	1			2			3		
		$\mu_{3}^{\prime }$	12	13	14	12	13	14	12	13	14
0.8	15	0.45470		0.44039	0.42707	0.45710	0.44270	0.42930	0.45940	0.44490	0.43150
	16	0.44807		0.43382	0.42056	0.45040	0.43600	0.42270	0.45270	0.43830	0.42490
	17	0.44203		0.42783	0.41463	0.44430	0.43000	0.41670	0.44660	0.43220	0.41890

1.0	15	0.47850		0.46330	0.44920	0.48090	0.46570	0.45150	0.48330	0.46800	0.45370
	16	0.47140		0.45630	0.44230	0.47380	0.45860	0.44450	0.47620	0.46090	0.44670
	17	0.46500		0.45000	0.43600	0.46730	0.45220	0.43820	0.46970	0.45450	0.44030

1.2	15	0.50280		0.48680	0.47190	0.50530	0.48920	0.47420	0.50780	0.49160	0.47650
	16	0.49530		0.47940	0.46460	0.49780	0.48170	0.46680	0.50020	0.48410	0.46910
	17	0.48850		0.47260	0.45790	0.49090	0.47490	0.46010	0.49330	0.47720	0.46230

For instance, the duration of vacations, the time taken for customers to arrive, and the duration of service for each customer have a significant influence on the system's ability to control congestion in the waiting area. Additionally, it has been observed that these factors lead to a reduction in the waiting time for patrons in the waiting hall; as the waiting area quantity ($E_{WH}$) decreases, so does the waiting time. Also, this analysis helps business tycoons to maintain an average stock level.

#### 6.2.2 Analysis of mean number of customers in the orbit

The impact of the parameters on the mean number of customers in the orbit is studied in this section. As with the waiting hall, the waiting time of orbital customers is also proportional to the number of customers in the orbit.

• The surge in arrival rates leads to extended customer wait times, resulting in a congested waiting area. This happens because once the waiting hall reaches its capacity, incoming customers are directed to the orbit. Consequently, in Table 5 $E_{O}$ increases with higher *λ*.

**Table 5:** Impact of *β*, *λ*, *η* and *θ* on *E*_*O*_.

*β*	*λ*	*η*	4			5			6		
		*θ*	0.06	0.08	0.10	0.06	0.08	0.10	0.06	0.08	0.10
4	0.6	23.16370		22.78740	22.55540	23.11680	22.73850	22.50760	23.07450	22.69470	22.46480
	0.8	23.90820		23.47400	23.15360	23.86240	23.42240	23.10260	23.82100	23.37610	23.05690
	1.0	24.50370		24.10500	23.72870	24.46130	24.05230	23.67500	24.42310	24.00490	23.62690

5	0.6	23.14890		22.77183	22.53897	23.10210	22.72310	22.49130	23.06000	22.67940	22.44860
	0.8	23.89142		23.45710	23.13616	23.84580	23.40570	23.08530	23.80460	23.35950	23.03970
	1.0	24.48463		24.08665	23.71012	24.44250	24.03410	23.65660	24.40450	23.98680	23.60870

6	0.6	23.12820		22.75020	22.51630	23.08163	22.70170	22.46879	23.03970	22.65820	22.42620
	0.8	23.86780		23.43360	23.11200	23.82252	23.38239	23.06135	23.78160	23.33640	23.01590
	1.0	24.45790		24.06100	23.68440	24.41608	24.00871	23.63121	24.37840	23.96170	23.58350
• Likewise, as the rate *θ* rises, customers transitioning from the orbit to the waiting area decreases. This leads to a reduction in $E_{O}$ in Table 5.
• Elevating the rate *η* prompts the server to transition from vacation mode to normal operation swiftly. This opens up space in the waiting area for customers from the orbit, which is clear from Table 5.
• An increase in replenishment rate *β* leads to a reduction in lead time. Consequently, customers do not experience prolonged waits in the waiting area during stock-out situations. This allows orbital customers to enter the waiting area, leading to a decrease in $E_{O}$, as shown in Table 5.
• The behavior of service rates remains consistent across both regular and vacation periods in this system. With increasing service rates, $E_{O}$ decreases because customers spend less time in the waiting area, as seen in Table 6, Table 7. This enables orbital customers to enter the waiting area.

**Table 6:** Impact of $\mu_{1},\;\mu_{1}^{\prime },\;\mu_{2}$ and $\mu_{2}^{\prime }$ on *E*_*O*_.

*μ*_1_	*μ*_2_	$\mu_{1}^{\prime }$	2			3			4		
		$\mu_{2}^{\prime }$	8	9	10	8	9	10	8	9	10
6	12	23.79600		23.62380	23.45460	23.61935	23.45049	23.28520	23.44630	23.28130	23.12020
	13	23.77640		23.60300	23.43290	23.59857	23.42878	23.26280	23.42460	23.25890	23.09740
	14	23.75700		23.58240	23.41150	23.57813	23.40753	23.24095	23.40350	23.23720	23.07520

7	12	23.77430		23.60100	23.43110	23.59660	23.42690	23.26110	23.42280	23.25720	23.09570
	13	23.75490		23.58050	23.40970	23.57610	23.40570	23.23920	23.40160	23.23540	23.07350
	14	23.73570		23.56040	23.38890	23.55610	23.38490	23.21800	23.38090	23.21420	23.05200

8	12	23.75279		23.57858	23.40795	23.57420	23.40380	23.23750	23.39970	23.23370	23.07190
	13	23.73361		23.55845	23.38711	23.55410	23.38310	23.21620	23.37900	23.21240	23.05030
	14	23.71474		23.53874	23.36680	23.53450	23.36280	23.19550	23.35880	23.19180	23.02940

**Table 7:** Impact of $\mu_{3},\;\mu_{3}^{\prime },\;\gamma_{1}$ and *γ*_2_ on *E*_*O*_.

*γ*_1_	*μ*_3_	$\gamma_{2}$	1			2			3		
		$\mu_{3}^{\prime }$	12	13	14	12	13	14	12	13	14
0.8	15	19.69611		19.80178	19.90151	19.71680	19.82290	19.92300	19.73750	19.84390	19.94440
	16	19.72772		19.83194	19.93029	19.74860	19.85330	19.95200	19.76950	19.87450	19.97370
	17	19.75567		19.85851	19.95554	19.77680	19.88000	19.97740	19.79790	19.90150	19.99930

1.0	15	19.90210		20.01180	20.11550	19.92260	20.03270	20.13680	19.94310	20.05360	20.15810
	16	19.93620		20.04440	20.14660	19.95690	20.06550	20.16810	19.97770	20.08670	20.18970
	17	19.96630		20.07310	20.17390	19.98730	20.09450	20.19570	20.00830	20.11590	20.21740

1.2	15	20.10670		20.22040	20.32800	20.12710	20.24120	20.34920	20.14750	20.26200	20.37040
	16	20.14340		20.25550	20.36160	20.16400	20.27660	20.38300	20.18470	20.29760	20.40450
	17	20.17590		20.28650	20.39110	20.19680	20.30780	20.41280	20.21770	20.32910	20.43450
• When perishable rates are raised, items in the system deteriorate rapidly, resulting in more frequent stock-out periods. This leads to an increase in the number of customers in the orbit, causing $E_{O}$ to rise in Table 7.

Studying the volume of customers in a designated area is a crucial practice for a business seeking to maximize its profitability. By examining the specific attributes associated with each parameter, business leaders can formulate effective strategies to cater to customers within and outside the establishment. This can lead to an augmented bottom line and an expanded market reach for the business.

In this context, two pivotal factors come into play: service time and lead time. These elements wield significant influence in reducing the wait times for customers in the designated area. For example, streamlining the service process and minimizing lead time can result in a more efficient and customer-friendly experience, which ultimately translates into higher customer satisfaction and increased revenue for the business.

#### 6.2.3 Analysis of mean number of failed items in the pooled space

In this section, the impact of the various parameters of the model over the mean number of replaceable items is analyzed through the Table 8, Table 9, Table 10. This analysis helps us to study the replacement of failed primary items in the system.

• With an uptick in arrival rate, more customers enter the system and make purchases before the product expires. Consequently, increasing *λ* leads to a decrease in the average number of failed items in Table 8.
• Similarly, elevating the *θ* rate leads to more sales before an item expires, resulting in a decrease in the average number of items that need replacement, which is shown in Table 8.
• When the vacation completion rate *η* rises, the duration of the vacation shortens, enabling the server to transition to regular working mode faster, thus facilitating more sales. Consequently, items are sold before they perish. Hence $E_{RI}$ decreases in Table 8.
• Increasing the rate *β* reduces lead time, increasing the mean inventory level for the primary commodity. This leads to an increase in the average number of replacement items in Table 8.
• As the perishable rate $\gamma_{1}$ increases, the lifespan of the primary commodity shortens, resulting in a rise in the average number of items in the pooled storage space, observable from Table 10.
• The service rates of the server during both working vacation and regular periods play a pivotal role in managing replacement items, with increased rates leading to higher sales. That is clear from the Table 9, Table 10.

Analyzing the volume of failed items in a communal storage space offers significant insights for efficiently overseeing perishable goods. This approach empowers businesses to comprehend the financial consequences, fine-tune their inventory management procedures, elevate customer satisfaction, adhere to regulatory requirements, and implement well-considered tactics to mitigate losses linked to items with a limited shelf life.

**Table 8:** Impact of *β*, *λ*, *η* and *θ* on *E*_*RI*_.

*β*	*λ*	*η*	4			5			6		
		*θ*	0.06	0.08	0.10	0.06	0.08	0.10	0.06	0.08	0.10
4	0.6	1.98718		1.97171	1.95663	1.98609	1.97046	1.95524	1.98511	1.96935	1.95400
	0.8	1.98241		1.96646	1.95116	1.98116	1.96508	1.94966	1.98003	1.96383	1.94831
	1.0	1.97831		1.96145	1.94580	1.97686	1.95992	1.94418	1.97556	1.95855	1.94273

5	0.6	2.09770		2.07990	2.06250	2.09650	2.07850	2.06090	2.09540	2.07720	2.05950
	0.8	2.09240		2.07400	2.05630	2.09100	2.07240	2.05460	2.08970	2.07100	2.05310
	1.0	2.08790		2.06840	2.05030	2.08630	2.06670	2.04850	2.08480	2.06510	2.04690

6	0.6	2.18210		2.16260	2.14370	2.18070	2.16110	2.14200	2.17960	2.15980	2.14050
	0.8	2.17640		2.15630	2.13710	2.17490	2.15470	2.13530	2.17350	2.15320	2.13370
	1.0	2.17160		2.15040	2.13070	2.16990	2.14850	2.12870	2.16830	2.14690	2.12700

**Table 9:** Impact of $\mu_{1},\;\mu_{1}^{\prime },\;\mu_{2}$ and $\mu_{2}^{\prime }$ on *E*_*RI*_.

*μ*_1_	*μ*_2_	$\mu_{1}^{\prime }$	2			3			4		
		$\mu_{2}^{\prime }$	8	9	10	8	9	10	8	9	10
6	12	2.08706		2.08815	2.08922	2.07450	2.07590	2.07720	2.06270	2.06430	2.06590
	13	2.08773		2.08882	2.08988	2.07520	2.07650	2.07780	2.06340	2.06500	2.06650
	14	2.08838		2.08946	2.09051	2.07580	2.07720	2.07850	2.06400	2.06560	2.06710

7	12	2.08261		2.08381	2.08498	2.07020	2.07170	2.07310	2.05857	2.06026	2.06189
	13	2.08337		2.08457	2.08573	2.07100	2.07240	2.07380	2.05931	2.06099	2.06261
	14	2.08411		2.08529	2.08644	2.07170	2.07310	2.07450	2.06002	2.06168	2.06329

8	12	2.07840		2.07970	2.08100	2.06620	2.06770	2.06920	2.05470	2.05650	2.05820
	13	2.07930		2.08060	2.08180	2.06700	2.06860	2.07010	2.05550	2.05730	2.05900
	14	2.08010		2.08140	2.08260	2.06780	2.06930	2.07080	2.05630	2.05800	2.05970

**Table 10:** Impact of $\mu_{3},\;\mu_{3}^{\prime },\;\gamma_{1}$ and *γ*_2_ on *E*_*RI*_.

*γ*_1_	*μ*_3_	$\gamma_{2}$	1			2			3		
		$\mu_{3}^{\prime }$	12	13	14	12	13	14	12	13	14
0.8	15	2.15682		2.16735	2.17751	2.15682	2.16735	2.17751	2.15682	2.16735	2.17751
	16	2.16250		2.17304	2.18321	2.16250	2.17304	2.18321	2.16250	2.17304	2.18321
	17	2.16778		2.17833	2.18852	2.16778	2.17833	2.18852	2.16778	2.17833	2.18852

1.0	15	2.23263		2.24074	2.24853	2.23263	2.24074	2.24853	2.23263	2.24074	2.24853
	16	2.23705		2.24515	2.25295	2.23705	2.24515	2.25295	2.23705	2.24515	2.25295
	17	2.24116		2.24925	2.25704	2.24116	2.24925	2.25704	2.24116	2.24925	2.25704

1.2	15	2.24621		2.25268	2.25890	2.24621	2.25268	2.25890	2.24621	2.25268	2.25890
	16	2.24978		2.25624	2.26245	2.24978	2.25624	2.26245	2.24978	2.25624	2.26245
	17	2.25309		2.25954	2.26574	2.25309	2.25954	2.26574	2.25309	2.25954	2.26574

In various business settings, metrics like arrival rate, retrial rate, and service rates play a crucial role in managing operations. For instance, in a restaurant, the arrival rate of customers determines how quickly tables must be cleared for the next diners. If not managed properly, this can lead to longer waiting times and unhappy customers. Similarly, in online retail, the retrial rate reflects how often customers attempt a purchase after encountering a technical issue. A high retrial rate may signal a website functionality problem. In a call center, the service rate indicates how many inquiries can be handled within a set time. If insufficient, this can result in frustrated customers waiting on hold or abandoning their calls. Monitoring and managing these metrics is essential for business efficiency and customer satisfaction.

#### 6.2.4 Analysis of fraction of time server is on working vacation mode

In this section, the characteristics of various parameters on the fraction of time server is on working vacation has been analyzed which is shown in the Table 11, Table 12, Table 13.

• In Table 11, $FT_{wv}$ decreases when we vary *η*. As the rate *η* rises, the server's vacation duration shortens, resulting in a decrease in $FT_{wv}$.
• Elevated rates of both *θ* and *λ* lead to higher eventual waiting times, subsequently decreasing the fraction of time allocated to working vacation as these rates increase, which is shown in Table 11.
• Increasing *β* leads to a gradual reduction in lead time. Consequently, an increase in the *β* rate diminishes the proportion of time spent on working vacation $FT_{wv}$. This impact is clearly shown in the Table 11.
• Table 12, Table 13 illustrate that any type of service enhancement results in an augmentation of the working vacation fraction $FT_{wv}$ due to the decrease in eventual waiting times.
• Similarly, in Table 13, elevating failure rates reduces the proportion of time allocated to working vacation, as higher failure rates lead to a decrease in inventory levels.

By examining the outcomes, we can observe how different factors impact $FT_{wv}$. This assessment enables companies to take proactive steps in resolving potential issues, improving the quality of service, and making sound financial choices. For example, allowing servers to work during their vacations can yield these advantages. However, it's crucial to take into account elements like labor regulations, the preferences of employees, and ensuring they receive sufficient rest periods to prevent overexertion. Moreover, it's imperative that such arrangements are optional rather than obligatory.

**Table 11:** Impact of *β*,*λ*,*θ* and *η* on *FT*_*wv*_.

*β*	*λ*	*η*	4			5			6		
		*θ*	0.06	0.08	0.10	0.06	0.08	0.10	0.06	0.08	0.10
4	0.6	0.1009		0.0891	0.0771	0.0990	0.0874	0.0756	0.0971	0.0858	0.0742
	0.8	0.0933		0.0814	0.0693	0.0915	0.0799	0.0680	0.0898	0.0784	0.0668
	1.0	0.0855		0.0736	0.0615	0.0839	0.0723	0.0604	0.0823	0.0710	0.0593

5	0.6	0.1005		0.0888	0.0768	0.0986	0.0871	0.0753	0.0967	0.0854	0.0740
	0.8	0.0928		0.0811	0.0691	0.0911	0.0795	0.0678	0.0894	0.0781	0.0665
	1.0	0.0851		0.0733	0.0613	0.0834	0.0719	0.0601	0.0819	0.0706	0.0591

6	0.6	0.1002		0.0885	0.0766	0.0983	0.0869	0.0752	0.0964	0.0852	0.0738
	0.8	0.0926		0.0809	0.0689	0.0908	0.0793	0.0676	0.0891	0.0779	0.0664
	1.0	0.0848		0.0731	0.0611	0.0832	0.0717	0.0600	0.0816	0.0704	0.0589

**Table 12:** Impact of $\mu_{1},\;\mu_{1}^{\prime },\;\mu_{2}$ and $\mu_{2}^{\prime }$ on *FT*_*wv*_.

*μ*_1_	*μ*_2_	$\mu_{1}^{\prime }$	2			3			4		
		$\mu_{2}^{\prime }$	8	9	10	8	9	10	8	9	10
6	12	0.07028		0.07029	0.07030	0.07143	0.07145	0.07146	0.07263	0.07264	0.07265
	13	0.07147		0.07148	0.07149	0.07266	0.07267	0.07268	0.07389	0.07390	0.07392
	14	0.07269		0.07270	0.07272	0.07393	0.07394	0.07395	0.07520	0.07521	0.07523

7	12	0.07031		0.07032	0.07033	0.07146	0.07147	0.07148	0.07265	0.07266	0.07267
	13	0.07149		0.07150	0.07151	0.07268	0.07269	0.07271	0.07392	0.07393	0.07394
	14	0.07272		0.07273	0.07274	0.07395	0.07396	0.07397	0.07523	0.07524	0.07525

8	12	0.07033		0.07034	0.07035	0.07148	0.07149	0.07151	0.07267	0.07269	0.07270
	13	0.07152		0.07153	0.07154	0.07271	0.07272	0.07273	0.07394	0.07395	0.07396
	14	0.07274		0.07275	0.07277	0.07397	0.07399	0.07400	0.07525	0.07526	0.07528

**Table 13:** Impact of $\mu_{3},\;\mu_{3}^{\prime },\;\gamma_{1}$ and *γ*_2_ on *FT*_*wv*_.

*γ*_1_	*μ*_3_	$\gamma_{2}$	1			2			3		
		$\mu_{3}^{\prime }$	12	13	14	12	13	14	12	13	14
0.8	12	0.08829		0.08840	0.08859	0.08830	0.08846	0.08860	0.08831	0.08850	0.08861
	13	0.09192		0.09209	0.09221	0.09193	0.09210	0.09226	0.09194	0.09212	0.09230
	14	0.09587		0.09605	0.09620	0.09589	0.09607	0.09624	0.09590	0.09609	0.09630

1.0	12	0.09278		0.09296	0.09312	0.09282	0.09300	0.09317	0.09284	0.09303	0.09319
	13	0.09653		0.09673	0.09690	0.09658	0.09678	0.09695	0.09660	0.09680	0.09697
	14	0.10062		0.10083	0.10102	0.10067	0.10088	0.10107	0.10070	0.10091	0.10109

1.2	12	0.09701		0.09721	0.09739	0.09707	0.09728	0.09746	0.09711	0.09731	0.09749
	13	0.10086		0.10107	0.10127	0.10093	0.10114	0.10134	0.10096	0.10117	0.10137
	14	0.10505		0.10528	0.10549	0.10512	0.10535	0.10556	0.10515	0.10539	0.10559

## 7 Managerial implications and insights

The findings of this inquiry have the potential to provide important techniques in the field of business administration. These insights have the potential to play a crucial role in providing guidance and assistance to managers who are directly engaged in the operations.

• **Optimal Replacement Strategy:** The comparison between the standard model without replacement and the model with replacement of failed items suggests that implementing a replacement facility can lead to a reduction in total expenditure for the business. This implies that managers should consider incorporating a replacement strategy as part of their operational approach to minimize costs.
• **Focus on Customer Attraction for Profit Maximization:** To enhance profitability, it is imperative for businesses to prioritize customer attraction. The research indicates that a multi-component demand approach leads to a higher influx of customers compared to a homogeneous arrival rate. Managers should, therefore, invest efforts in diversifying their product or service offerings to cater to a broader customer base.
• **Leveraging Service Rates for Efficiency:** The analysis consistently highlights the positive impact of service rates. This suggests that managers should focus on optimizing and possibly increasing service rates wherever applicable, as it can improve operational efficiency and customer satisfaction.
• **Managing Failed Items in Pooled Storage:** The numerical analysis of the expected number of failed items in pooled storage underscores the influence of the multi-component demand rate on this metric. Managers should closely monitor and manage this aspect to effectively control the number of failed items, potentially leading to cost savings and improved customer service.
• **Optimizing Service, Vacation, and Reorder Rates:**Increasing the service rate, vacation completion rate, and reorder rate directly leads to a reduction in the number of customers in the waiting hall. This implies that managers should focus on optimizing these operational parameters to enhance the flow of customers through the system. This can lead to shorter wait times and improved customer satisfaction.
• **Mitigating Congestion in the Orbit:** The study indicates that congestion in orbit can be alleviated by taking specific actions, such as increasing the reorder rate, vacation completion rate, and retrial rate. This suggests that managers should focus on implementing strategies to enhance these operational parameters, leading to smoother operations and improved customer experiences so that the customers outside the business can benefit.

Overall, these findings provide valuable insights that managers can leverage to make informed decisions aimed at optimizing operational efficiency, reducing costs, and enhancing customer satisfaction in their respective businesses.

## 8 Conclusions, findings, limitations, and direction for future implications

### 8.1 Conclusion

This study examines the replacement of a failed primary commodity in a two-commodity retrial QIS that incorporates multi-component demand rates and vacation interruptions. A level-dependent quasi-birth-and-death process was constructed in order to analyze the model analytically. Initially, the stability condition was established in order to get the steady-state probability vector. Subsequently, Neuts and Rao's truncation approach is used to estimate the joint probability vector in the steady state. Additionally, many performance indicators for system characteristics are constructed. The quantitative investigation of the system facilitates the examination of the attributes and efficacy of the model. The comparison between the system that incorporates replacement and the system that does not include replacement demonstrates that the one with replacement operates with more efficiency, hence minimizing potential business losses caused by item failure. When comparing homogeneous and multi-component demand, the model that includes multi-component demand rates has a greater ability to attract users to the system, resulting in a higher likelihood of achieving profit gains. The investigation focuses on the analysis of the expected number of customers present in both the waiting hall and the orbit, taking into consideration all of the variables involved. A numerical investigation is conducted to examine the impact of parameters on the replacement of defective items. The examination of trade-offs between normal and working vacation modes, as well as the server's decision-making process, has significant importance in optimizing service efficiency and resource utilization.

### 8.2 Findings

• The comparison between the standard model without replacement option and replacement of failed items reveals that the replacement facility minimizes the total expenditure in business.
• In the business environment, a crucial aspect of increasing profit is encouraging customer attraction. Multi-component demand increases the customer arrival rate more than the homogeneous arrival rate.
• The service rates are influenced favorably in all the numerical studies.
• Numerical analysis of the expected number of failed items in the pooled storage shows that the multi-component demand rate controls it.
• The congestion in the orbit can be decreased by increasing the reorder rate, vacation completion rate, and retrial rate.

### 8.3 Limitations and direction for future implications

There are a few limitations within our study that may be addressed in future research.

• The model is considered for single server facility only. This work can be extended in the future with heterogeneous multi-server facilities.
• Replacement facility is applied only to the primary item as the second commodity is complementary. However, in the future, replacement ideas can be applied to both commodities.
• For the first commodity, (s, Q) ordering policy is adopted, and instantaneous ordering policy is adopted for the second commodity. However, individual ordering policy can be studied.
• The service times follow an exponential distribution. Phase-type distribution can be adopted in future works.
• In this study, numerical analysis of various key system performance measures is conducted. A real-life case study of the model can be addressed in the future.

## CRediT authorship contribution statement

**K. Jeganathan:** Writing – original draft, Investigation, Formal analysis, Conceptualization. **V. Anzen Koffer:** Writing – original draft, Software, Resources, Methodology. **K. Lakshmanan:** Visualization, Validation, Software, Resources. **K. Loganathan:** Writing – review & editing, Resources, Project administration, Funding acquisition. **Mohamed Abbas:** Writing – review & editing, Project administration, Investigation, Funding acquisition. **A. Shilpa:** Supervision, Software, Resources, Methodology.

## Declaration of Competing Interest

The authors declare that they have no conflicts of interest.

## Data Availability

The data will be made available on request.
